# Lagged recovery of fish spatial distributions following a cold-water perturbation

**DOI:** 10.1038/s41598-021-89066-x

**Published:** 2021-05-04

**Authors:** M. D. Robertson, J. Gao, P. M. Regular, M. J. Morgan, F. Zhang

**Affiliations:** 1 Centre for Fisheries Ecosystems Research, Fisheries and Marine Institute of Memorial, University of Newfoundland, P.O. Box 4920, St. John’s, NL A1C 5R3 Canada; 2 Fisheries and Oceans Canada, Northwest Atlantic Fisheries Centre, 80 East White Hills Rd., P.O. Box 5667, St. John’s, NL A1C 5X1 Canada

**Keywords:** Population dynamics, Animal migration, Ecology, Climate-change ecology

## Abstract

Anomalous local temperature and extreme events (e.g. heat-waves) can cause rapid change and gradual recovery of local environmental conditions. However, few studies have tested whether species distribution can recover following returning environmental conditions. Here, we tested for change and recovery of the spatial distributions of two flatfish populations, American plaice (*Hippoglossoides platessoides*) and yellowtail flounder (*Limanda ferruginea*), in response to consecutive decreasing and increasing water temperature on the Grand Bank off Newfoundland, Canada from 1985 to 2018. Using a Vector Autoregressive Spatiotemporal model, we found the distributions of both species shifted southwards following a period when anomalous cold water covered the northern sections of the Grand Bank. After accounting for density-dependent effects, we observed that yellowtail flounder re-distributed northwards when water temperature returned and exceeded levels recorded before the cold period, while the spatial distribution of American plaice has not recovered. Our study demonstrates nonlinear effects of an environmental factor on species distribution, implying the possibility of irreversible (or hard-to-reverse) changes of species distribution following a rapid change and gradual recovery of environmental conditions.

## Introduction

Climate change can affect the range of physiologically suitable habitats of species, leading to shifts in their spatial distributions across terrestrial, freshwater, and marine ecosystems^1–3^. For example, there is increasing evidence of unidirectional, often poleward, shifts of marine organisms in response to changes in the distribution of their thermal habitat^3–5^. Shifts in the distribution of thermal habitat can influence species spatial distribution via direct effects on animal physiology and phenology^6–8^, and indirect effects on biotic interactions^9^. For instance, when temperature exceeds a mobile organisms’ physiological threshold, the organism will move towards habitat with tolerable thermal conditions^10^. Although many studies focus on unidirectional shifts in species distributions due to environmental change at regional or global scales^2^, organisms generally experience and respond to the local environment which may not show monotonic variations^11,12^. As a result, species distributions may exhibit more complex dynamics in response to fluctuating environmental conditions.

**Figure 1:**
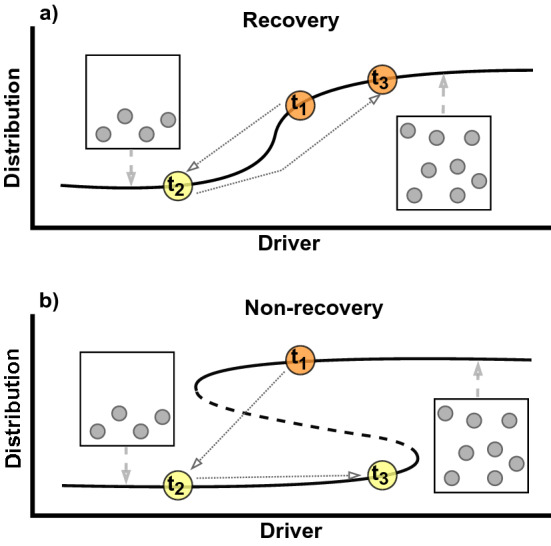
Conceptual diagram of spatial distribution recovery and non-recovery. The curved lines represent how an organism’s spatial distribution will change with an environmental driver where, (**a**) is a distribution that recover’s when the environmental driver returns to previous conditions and (**b**) is a distribution that does not recover when the environmental driver returns to previous conditions. The S-shape in (**b**) represents an unstable equilibrium between distributional states, where a distribution above the dashed line will be driven to the upper asymptote and distributions below the dashed line will be driven the lower asymptote (similar to the concept of hysteresis in ecosystem states^70^). The colored circles on the lines represent three snapshots in time (t_1_–t_3_) that are meant to represent the same environmental driver conditions between panels a and b, and the dotted grey arrows represent the transition between each snapshot. The circles within boxes represent the snapshots of the spatial states for the organism’s distribution. Figure made using the software diagrams (diagrams.net).

Anomalous local temperature and extreme events in particular can cause rapid changes in species distributions^13–15^. For example, a marine heat wave accelerated the poleward shift in the spatial distribution of a temperate marine fish (*Silago schomburgkii*) in Western Australia^16^. Following anomalous events, environmental conditions typically show a gradual return to previous levels. However, few studies have tested whether species distribution can recover following returning environmental conditions (Fig. 1; i.e. whether species distribution responds linearly to environmental factors). This is especially important to natural resource management because irreversible (or hard-to-reverse) changes in species distributions may lead to spatial mismatch with management/conservation areas, increasing the risks of local overexploitation or underutilization of natural resources^17^. Furthermore, understanding distributional change and recovery following fluctuating environmental conditions is important for predicting species response to climate change beyond the current focus on unidirectional trends^14^.

**Figure 2:**
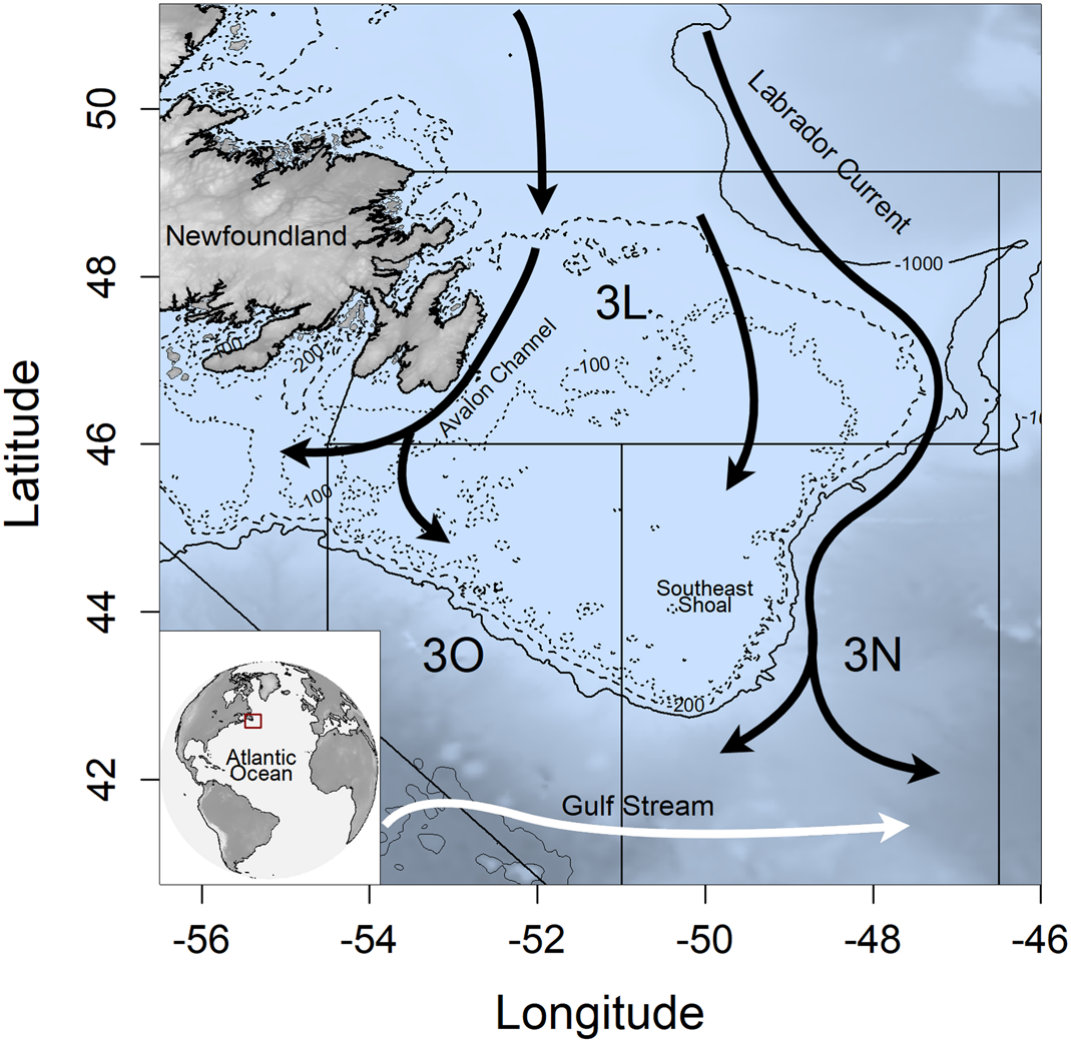
Bathymetry and dominant currents of the Grand Bank region/North Atlantic Fisheries Organization (NAFO) divisions 3LNO. Bathymetric contours shown for 100 (dotted lines), 200 (dashed lines), and 1000 m (solid lines). Approximate pathways for the Labrador Current represented by black arrows and the Gulf Stream represented by white arrows. Inset in the bottom left shows the position of the study area (red rectangle) in reference to the rest of the Atlantic Ocean. Figure made using R (version 3.6.2. https://www.r-project.org/)^69^ and the packages sp^71^, rgdal^72^, raster^73^, marmap^74^, grid^69^, gridbase^75^, and maptools^76^. Bathymetry and coastal shapefiles were taken from the package marmap and the NAFO division shapefile was taken from NAFO (https://www.nafo.int/Data/GIS). Arrows indicating current directions were added in the software diagrams (diagrams.net).

In this study, we aim to test for change and recovery of the spatial distributions of two flatfish populations, American plaice (*Hippoglossoides platessoides*) and yellowtail flounder (*Limanda ferruginea*), in response to consecutive decreasing and increasing temperatures on the Grand Bank off Newfoundland, Canada. The Grand Bank is an underwater plateau located at the confluence of the Labrador current and Gulf stream in the northwestern Atlantic ocean (Fig. 2). This ecosystem once maintained one of the world’s most productive and valuable commercial fisheries^18^, however, in the mid-1990s, the biological community shifted from groundfish-dominated to being dominated by lower trophic level fishes and invertebrates^19^, which was hypothesized to be associated with prolonged intensive fishing and a period of anomalously cold ocean temperature^20^. Ocean temperature affects population distribution via density-independent effects^21^ and fishing mainly affects population distribution via density-dependent processes (i.e. changes in total population size)^22,23^. For example, density-dependent processes are expected to drive population distributions to expand towards marginal habitats when population density increases and contract towards core habitats when population density declines^24–26^. Therefore, to test for the change and recovery of spatial distributions in response to changing water temperature, we will (1) derive spatiotemporal variations of bottom water temperature on the Grand Bank, 2) model the spatiotemporal changes in yellowtail flounder and American plaice distributions, (3) account for density-dependent effects of population size on spatial distribution, and (4) test for a change and recovery of population distribution following a change and recovery in bottom water temperature. More detailed descriptions of each method can be found in “Materials and Methods” section.

## Results

### Bottom water temperature interpolation

Bottom water temperature was spatially heterogeneous on the Grand Bank. The spatial distribution of bottom water temperatures was relatively consistent throughout the study period with the southern and northeastern sections of the bank being the warmest (see Fig. 2 for geographical reference) and the interior, northern half of the bank and Avalon channel being the coldest (Fig. 4a; Figs. S1–S5). From 1977–1990, temperature throughout the bank declined and eventually reached a minimum in 1990. During 1989–1991, the Grand Bank had large areas, specifically in the interior, northern half of the bank, where water temperature was lower than any location on the bank during any of the years examined here (< − 1 °C; Fig. 4). Following this cold period, water temperature increased throughout the bank and returned to and even exceeded the levels observed in the late-1970s and early-1980′s. We examined the spatiotemporal correlation of temperature across space through time (Fig. S6), which provided further evidence that the spatial pattern of water temperature was similar through time. Therefore, this relatively constant spatial distribution of bottom water temperature is used as spatial reference for changes in fish distributions in later analyses.

### VAST estimates

Both yellowtail flounder and American plaice on the Grand Bank underwent distributional changes between 1985 and 2018 (Figs. S7 and S8). Yellowtail flounder were initially distributed throughout the interior of the Grand Bank. Their spatial distribution contracted and moved ~ 80 km south and ~ 20 km west (Figs. S7 and S9d) during the cold period between 1985 and 1995, and re-expanded northeastwards across the interior of the bank afterwards (Fig. S9d). The contraction and re-expansion of yellowtail flounder, as measured by the effective area occupied, was positively correlated with changes in the population’s biomass which declined and then recovered (ρ = 0.73, *p* value < 0.01; Fig. S9c). American plaice were distributed throughout the entire Grand Bank in the mid-1980s. Their distribution contracted and shifted ~ 200 km southwards during the cold period (Figs. S8 and S9b). Since contracting southwards, American plaice have not re-distributed to their previous location 200 km north, instead they have moved 50 km northeast (Fig. S9b). Further, the contraction of the American plaice distribution, as measured by the effective area occupied, was correlated with changes in the population’s biomass which declined and have yet to recover (ρ = 0.73, *p* value < 0.01; Fig. S9a).

### Density-dependent habitat selection

A linear relationship without evidence of model misfit between total population biomass and local population density (than population density at each of the fifty locations estimated in the VAST) was detected for American plaice (Figs. S10 and S12) but not for yellowtail flounder (Figs. S11 and S13). After fitting a linear model to the total and local population size, the model had a relatively high R^2^ (0.38) and the residuals showed a normal distribution for American plaice (Fig. S12). Whereas, for yellowtail flounder, the model had a low R^2^ (0.07) and the residuals had a bimodal distribution (Fig. S13). Density-dependent habitat selection is expected to yield differential growth rates in optimal and marginal habitats as population size increases^27^. The relationship observed for American plaice was indicative of a proportional response in density to increasing population size, while yellowtail flounder’s bimodal residuals and low R^2^ indicated locally varying relationships between local and global biomass. (see Testing for density-dependent habitat selection for further rationale of this methodology). Therefore, these analyses indicated that density-dependent habitat selection was strong for yellowtail flounder yet not detectable in American plaice. As a result, we were able to test for the response of American plaice’s spatial distribution to changing water temperature using the local density estimates from VAST directly.

**Figure 3:**
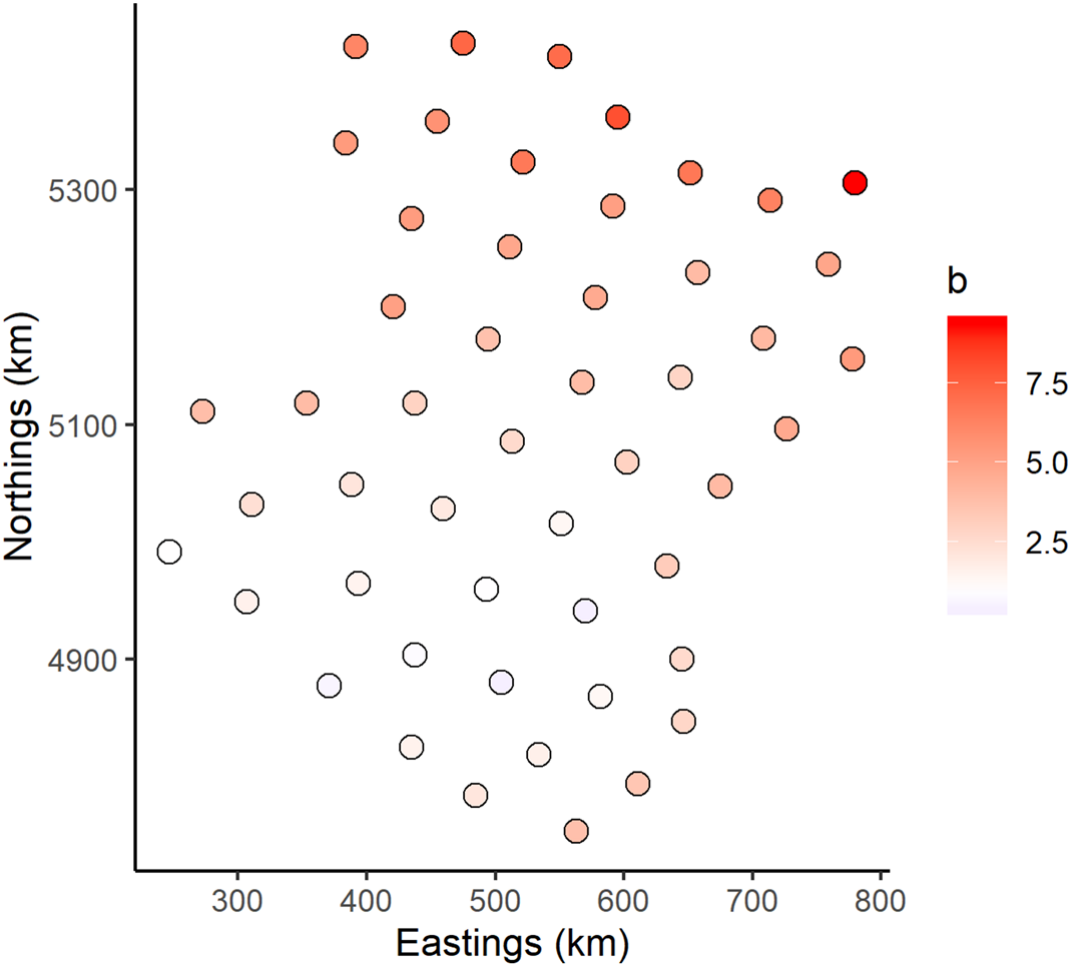
The estimate of the slope (exponent) between local and total biomass (b_k_) from the knot specific exponential models comparing yellowtail flounder local density based on total biomass across the fifty knots in the VAST. Red circles represent a b_k_ > 1 which indicate large local biomass changes with total biomass changes (i.e. marginal habitat), white circles represent a b_k_ = 1, and blue circles represent a b_k_ < 1 which indicate that local biomass is stable with changes in total biomass (i.e. ideal habitat). Figure made using R (version 3.6.2. https://www.r-project.org/)^69^ and the packages sp^71^ and ggplot2^77^.

Using an exponential mixed-effects model, we identified local variability in the relationship between local density and total population biomass for yellowtail flounder (Figs. S14 and S15). The model indicated that the preferred habitat for yellowtail flounder existed throughout the southern Grand Bank with ideal habitat (b_k_ < 1) on the southeast shoal and the most marginal habitat on the northern edge of the Grand Bank (b_k_ > 1; Fig. 3). The spatiotemporal residuals from these models were grouped in space and time (Fig. S16) indicating that density-dependent habitat selection alone could not fully explain the shifts in spatial distribution for yellowtail flounder. Therefore, we used these residuals to test for the effects of water temperature on yellowtail flounder’s spatial distribution.

### Distribution: temperature correlation

**Figure 4:**
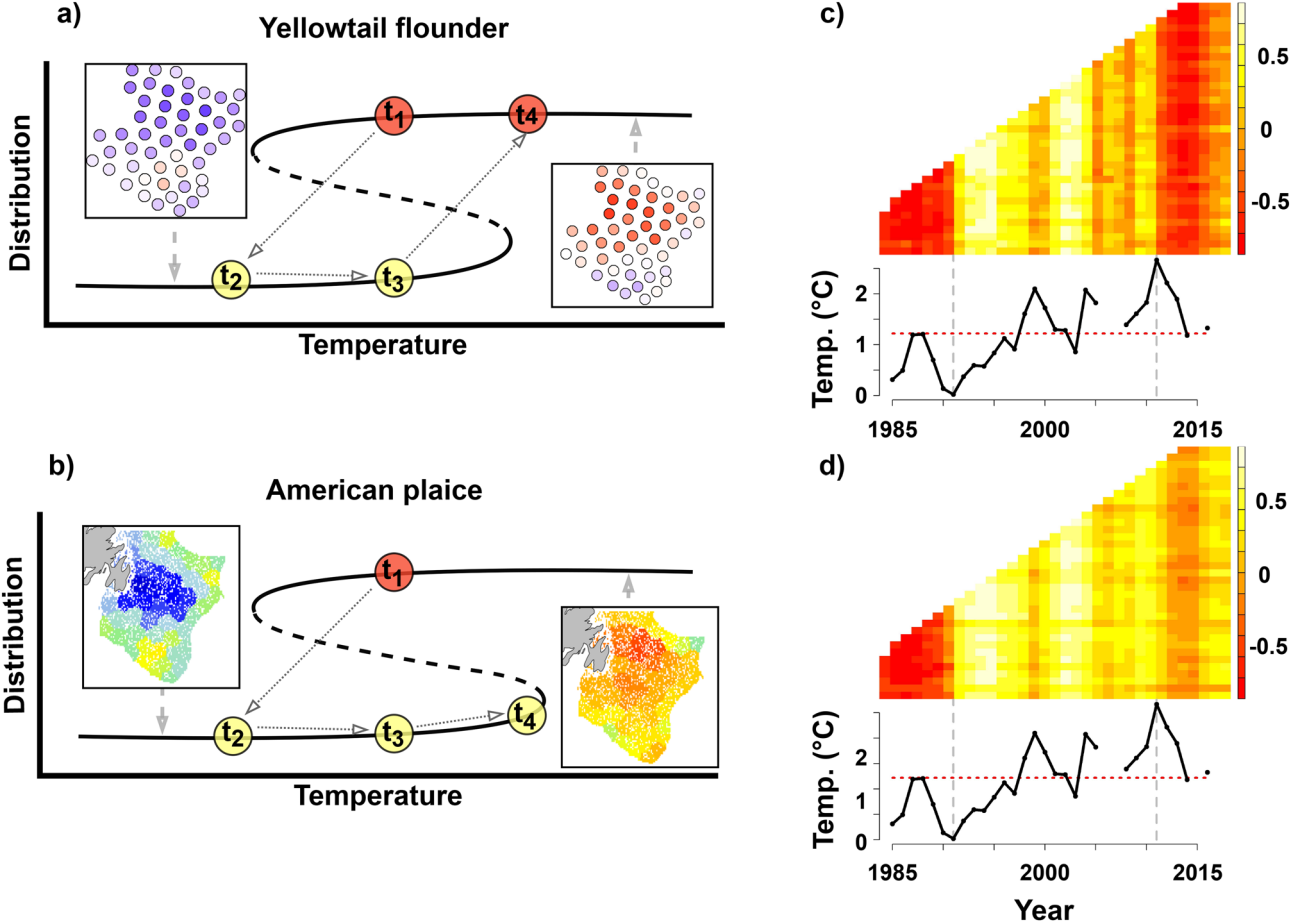
Diagram of spatial distribution recovery results, format based on Fig. 2. Line drawings for (**a**,**b**) represent the theoretical pattern that matches the observations for each species. t_1_ and t_3_ represent snapshots in time with equal temperature, while t_4_ represents a snapshot with a higher temperature. Maps for (**a**,**b**) represent snapshots that define each spatial state, where the maps for (**a**) represent the residuals (blue = negative, red = positive) of the density-dependent model for yellowtail flounder (see Fig. S16) and maps for (**b**) represent the spatial distribution (blue = low density, red = high density) of American plaice from the Vector Autoregressive Spatiotemporal model [VAST] (see Fig. S8). Finally, (**c**,**d**) is the correlation analysis of spatial distributions (y-axis) and bottom water temperature distributions (x-axis) over time (yellow = high correlation, red = negative correlation) along with the mean annual temperature trend on the Grand Bank shown beneath the correlation plots (dashed grey lines represent the minimum [1991] and maximum [2011] mean temperatures; dotted red line represents the mean temperature over the entire time-series). High correlation in panels (**c**,**d**) indicates that areas with high density occur in areas with warmer temperatures and negative correlation indicates that areas with high density occur in areas with cold temperatures. Figure made using R (version 3.6.2. https://www.r-project.org/)^69^ and the packages sp^71^, ggplot2^77^, VAST^78^, raster^73^, fields^79^, RColorBrewer^80^ and the software diagrams (diagrams.net).

Yellowtail flounder’s spatial distribution recovered after water temperature returned and exceeded previous levels. The residuals from the density-dependent habitat selection model were positive in the northern Grand Bank prior to the cold period, indicating more fish than expected by density-dependence alone (Fig. S16). Following the cold period, the residuals in the north became negative, and these negative values persisted when warm temperatures returned throughout the Grand Bank (Fig. S16). The residuals did not become positive again until 2010, the year with the warmest mean temperature on record for the Grand Bank (~ 2.5 °C). Using the correlation between these residuals and the spatial distribution of temperature, we observed an abrupt shift from negative to positive correlation in the coldest year (1992; Fig. 4d). The positive correlation between temperature and yellowtail flounder distribution persisted until the warmest year (2010) which marked a second abrupt shift to negative correlation, which has mostly persisted since (Fig. 4d). Therefore, there was evidence of multiple distribution states for the same environmental conditions (temperature), where the switch between states corresponded with particularly cold and particularly warm temperatures.

Non-recovery in spatial distribution was observed for American plaice. American plaice distribution was negatively correlated with spatial temperatures prior to 1991 but became positively correlated in the years after 1991 (Fig. 4e). Unlike yellowtail flounder, the distribution of American plaice has yet to be negatively correlated with temperature since 1991 (Fig. 4e). Therefore, the American plaice population has evidence of two distinct spatial states: an early state with a wide distribution throughout the bank that was negatively correlated with temperature, and a recent state with a more contracted and southerly distribution that is positively correlated with temperature. Similar to yellowtail flounder, both states have existed during years with similar temperatures and the shift from the early state to the recent state corresponded with the timing of the coldest years. However, unlike yellowtail flounder, American plaice have yet to return to their previous distribution even when temperature became warmer than the levels before the cold period.

## Discussion

We identified evidence that yellowtail flounder and American plaice have undergone shifts in their distribution over the past 35 years. These shifts differed in scale, where American plaice contracted and shifted their distribution hundreds of km’s, while yellowtail flounder contracted and had relatively small changes in their central location. Furthermore, both species experienced a population collapse in the early 1990s but only yellowtail flounder has recovered since. To account for the potential effects of changing population size on distribution, we examined the role of density-dependent habitat selection which appeared to influence the distributional shift for yellowtail flounder but not American plaice. Past studies have reached the same conclusions for both populations^28,29^. However, density-dependent habitat selection did not fully describe the distributional changes for yellowtail flounder. Furthermore, American plaice have yet to return to the northern Grand Bank where their distribution was initially centered, despite the Grand Bank warming in recent years.

By testing for changes in the correlation between spatial temperature and the spatial distribution of populations, we detected periods of change and non-recovery in the distribution of yellowtail flounder and American plaice. The cold-period that we observed from 1990 to 1992 matched previous observations of below average water temperatures on the Grand Bank that were accompanied by record high ice extent and record low salinity^19,30^. Our results suggest that anomalous temperatures can elicit long-term changes, which may represent alternative distributional states that can be difficult to reverse^31^. In both species, local fish density in the northern Grand Bank has shown the smallest amount of recovery. In addition, yellowtail flounder returned to their initial distributional state following a recent period of warm temperatures in 2011, while American plaice did not. This indicates that although both species exhibited a lack of recovery following the same cold period (i.e. a temperature threshold) they appear to have unique temperature thresholds that must be reached to return to their initial states. In the following paragraphs, we will discuss the possible ecological mechanisms, management implications, and future research directions of non-recovery of spatial distributions following an anomalous climate event.

Non-recovery of spatial distributions may be caused by spatially varying environmental effects acting on local populations (i.e. lower-level effects). The initial effects of a disturbance on local populations will depend on the level of exposure to the disturbance^32^. American plaice have historically preferred colder temperatures than yellowtail flounder (Figs. S26–S28), so, compared to yellowtail flounder, American plaice were more distributed in the northern half of the Grand Bank. The colder-water disturbance was strongest in the northern part of Grand Bank, causing American plaice to have higher level of exposure to disturbance than yellowtail flounder. For example, although the colder-water disturbance led to shifting thermal habitats for both species, American plaice’s median occupied temperature increased by approximately 2 °C following the cold period, while yellowtail flounder’s increase was relatively small (approximately 0.5 °C) in comparison (Fig. S28). The combined effects of this increased level of exposure may affect population recovery, since the northern component of the American plaice population was historically a major source of recruitment success^33^. In addition, the magnitude of the effect of disturbance on local density is dependent on the resilience of local populations^34^. Resilience to major disturbances may be decreased in populations that have previously been affected by persistent minor disturbances and, as such, populations that have been exposed to anthropogenic pressures (e.g. prolonged fishing pressure^23^) may experience greater risk of prolonged change. Furthermore, reduced population density and productivity in certain locations can affect metapopulation dynamics^35,36^. Connectivity and productivity of sub-populations within a metapopulation are necessary for the persistence of sub-populations through time, and spatially heterogeneous disturbances have the ability to fragment habitat and reduce productivity^37,38^. Finally, spatial differences in individual physiology^39,40^ and/or demographic traits^41,42^ could impact the recovery of a population. If the organisms that survived the disturbance lack the physiological or demographic traits that permitted survival or reproductive success in the disturbed areas, then re-colonization of those areas would be reliant on demographic or physiological changes.

Non-recovery of spatial distributions could also be caused by spatially reorganized community structure (i.e. higher-level effects)^31^. Biotic interactions (e.g. competition, predation) define community structure and these interactions are dependent on the abundance and presence (i.e. spatial overlap) of interacting species^43^. Therefore, spatially heterogeneous disturbances can initiate unique successional pathways (i.e. changes in an ecological community following a disturbance) that reorganize local community structure^44,45^. For example, the cold period on the Grand Bank may have promoted population growth of previously non-dominant species (e.g. snow crab^46^) while also reducing the abundance of dominant species (e.g. Atlantic cod) in specific locations. This shift in dominance can modify biotic interactions in those locations and force long-term community reorganization^47^. These local reorganizations would create a spatially heterogeneous interaction network that may impede recovery by modifying the spatial availability of suitable habitat for a particular species^48^. Such changes have been previously observed in plant communities and were proposed as a potential leading indicator for regime shifts^48^. Additionally, we observed distributional shifts of both species (more dramatically for American plaice) towards warmer waters during a warming period, rather than moving to maintain prior thermal conditions as would generally be expected^2,4^. Regardless of the direction, distributional shifts can modify ecological communities by forcing the development of novel interactions between species that did not previously overlap^49^. Therefore, non-recovering distributions may be indicative of drastic changes in local community ecology, despite only being an observable pattern for a few species.

It is important to note that the non-recovery of distributions discussed here may be only partially related to changes in temperature. For example, changes in the ecological community on the Grand Bank are hypothesized to be the result of both fishing and environmental drivers^19^. However, due to a lack of spatiotemporal time-series of fishing pressure (see Fig. S17 for spatially aggregated time-series), we were unable to directly examine the influence of fishing on population distributions. Despite not being able to account for fishing pressure directly, we were able to assess the relationship between changes in population size (one effect of fishing pressure) and spatial distribution. However, it is also worth noting that given the observational nature of the data used, we were only able to test for the effects of population size through the mechanism of density-dependent habitat selection. It is possible that population size could interact with changing temperatures or affect distribution through other mechanisms. Being unable to observe all potential drivers is an inevitable challenge of empirical study in natural systems, since it is impossible to collect data on all aspects of an ecosystem. Therefore, future research on long-term changes in spatial distributions would benefit from experimental and theoretical examinations capable of controlling for additional drivers. Finally, the observed pattern is dependent on the scale of observation. For example, if our study had examined the northern and southern Grand Bank separately, we may conclude that American plaice in the northern section of the bank had not recovered while the southern section of the bank had. Although we cannot yet identify the mechanisms responsible for the distribution shift, an ability to identify the pattern of change is a necessary first step towards producing plausible, testable hypotheses that will inform conservation and resource management.

Modified spatial distributions of species can affect conservation and resource harvesting strategies. Distributional shifts have been observed as a response to changing climate^2,4^ and the identification of these shifts has led to the development of protected areas and management plans that estimate future distributions using species distribution models^50^. Although we observed that species on the Grand Bank initially tracked local climate (cooler temperatures) by shifting their distributions southwards, the lack of recovery has maintained the southerly distributions despite the return of warm temperatures. This potentially irreversible shift is problematic because distributional shifts in unexpected directions (e.g. towards warm waters during a warming period) and non-recovery of distributions following environmental recovery may not be predicted with species distribution models^51^. These unexpected changes will affect the efficacy of protected areas developed for areas with presumed stationary landscape structure or protected areas that shift with climate^52^. Therefore, the development of connected, protected area networks throughout a heterogeneous environment may be the best strategy for buffering the effects of disturbance and minimizing the risk of non-recovery^53^. Additionally, the non-linear response of spatial distributions provides evidence for the necessity of using various ecological indicators (including spatial distributions) when assessing the status of an ecosystem or a population^54^. Population status is often assessed based on population change within specific management boundaries, and unacknowledged distributional shifts across a management boundary may impact the perceived population size and/or vital rates (growth, reproduction, mortality) that are used for the development of management strategies^17,55^. We have only examined species within their management boundaries here and therefore cannot assess whether this problem may exist for populations in the Northwest Atlantic. Successful management of species affected by non-recovering spatial distributions will require a recognition of the change, as well as a determination of the effects of the spatial change on estimates of population status and recovery potential.

We have used the concept of non-recovery (i.e. non-linear response) to describe the observed patterns of spatial change, and have provided various lines of evidence for what may be causing this pattern and why it merits further investigation. Future studies should continue to examine spatial distribution shifts following anomalous events to identify if non-recovery is a widespread phenomenon. Additionally, further study on the role of biotic interactions in shaping the distribution of species will improve our understanding of how modified feedbacks may promote persistent changes in spatial distributions^56,57^. Finally, continued exploration of the mechanisms that erode spatial population structure are necessary to develop conservation and management strategies that will minimize the possibility of degrading spatial distributions^23^. In conclusion, by analyzing the non-recovery of species distributions following anomalous climate events, we will gain a better understanding of the causes and consequences of prolonged distributional changes, the diversity of responses to climate change, and be able to better identify ways to mitigate their negative socio-ecological effects.

## Materials and methods

### Spatiotemporal temperature interpolation

We examined annual stratified random, bottom trawl surveys, conducted in the spring (April–June) by the Canadian Department of Fisheries and Oceans (DFO), on the Grand Bank off Newfoundland in Northwest Atlantic Fisheries Organization (NAFO) divisions 3LNO from 1977 to 2018^58,59^. We excluded data from 1981, 1983, and 1984 due to poor spatial survey coverage. We used ordinary kriging to estimate annual temperatures throughout the Grand Bank with the automap package^60^ in R (see Figs. S1–S5). To accomplish this, we fit a variogram to the annual raw temperature data (200–400 samples year^-1^) and then predicted temperatures across the Grand Bank using the weighted least squares method to fit a spherical model to the sample variogram with a nugget effect, considering all observations for the kriging neighborhood^61^. We sampled the annual temperature estimates using the same knot locations used in the VAST model (see Spatiotemporal model section) to allow direct spatial comparisons between fish density and bottom-water temperature. Finally, we estimated a mean annual time-series of bottom-water temperature from the same knot locations described above to permit temporal comparisons between shifts in spatial distribution and bottom-water temperature.

### Spatiotemporal model

We used fish biomass data from the aforementioned DFO research vessel surveys from 1985 to 2018 to model fish distribution changes over time. We excluded data collected prior to 1985 due to incomplete survey coverage and differences in gear. These data were modeled using VAST, a modeling platform to assess how the distribution of species/communities have changed over time^62^. VAST is capable of predicting density across locations *s*, and time intervals *t* for multiple categories *c* (here, *c* is species)^63^*.* VAST model predictions are made across a pre-specified number of locations (here, 50 locations, referred to as knots) within a Gaussian Markov Random Field, such that a prediction at any location is equal to its value at the nearest location. A variety of recent research has shown that VAST is capable of providing sound spatio-temporal advice to fisheries management, specifically for estimating indices of abundance, distribution shifts, and range expansion/contraction^62,64^.

The parameterization used here involves a delta model to separately model encounter probability *p* and biomass density via positive catch rates *r*:

1 $$\Pr \left( {b_{i} = B} \right) = \left\{ {\begin{array}{*{20}l} {1 - p_{i} } \hfill & {\quad {\text{if}}\;{\text{B}} = 0} \hfill \\ {p_{i} \times Gamma\left( {B;\log \left( {r_{i} } \right),\sigma_{b}^{2} \left( c \right)} \right)} \hfill & {\quad {\text{if}}\;{\text{B}} > 0} \hfill \\ \end{array} } \right.$$

where *b*_*i*_ is the sampled biomass for each sample *i.* We specifically used a Poisson-link delta model which is proposed to be more biologically interpretable than the conventional delta model because it correlates predicted encounter probability and positive catch rates based on a joint dependence on group biomass density^65^. Encounter probability *p*_*i*_ assumes that individuals are randomly distributed in the sampling area and the probability of encountering at least one fish is modeled as

2 $$p_{i} = 1 - \exp \left( { - \alpha_{i} \times \exp \left( {p_{1} \left( i \right)} \right)} \right)$$

where *α*_*i*_ is an offset for the area swept by the bottom trawl and *p*_1_ is an encounter probability linear predictor that is described below. Positive catch rate *r*_*i*_ is then defined as

3 $$r_{i} = \frac{{a_{i} \times {\text{exp}}\left( {p_{1} \left( i \right)} \right)}}{{p_{i} }} \times {\text{exp}}\left( {p_{2} \left( i \right)} \right)$$

where $p_{2}$ is a positive catch rate linear predictor that is described below.

Both the encounter probability and positive catch rates were estimated spatiotemporally using separate linear predictors, where encounter probability is modeled using a logit-link and

4 $$p_{1} \left( i \right) = {\text{logit}}\left[ {p\left( {s_{i} ,c_{i} ,t_{i} } \right)} \right] = \gamma_{p} \left( {c_{i} t_{i} } \right) + \omega_{p} \left( {s_{i} c_{i} } \right) + \varepsilon_{p} \left( {s_{i} c_{i} t_{i} } \right)$$

where *s* is the location, and *t* is the time of the sample. $\gamma_{p} \left( {c_{i} t_{i} } \right)$ is an intercept for temporal encounter probability for each category, $\omega_{p} \left( {s_{i} c_{i} } \right)$ estimates spatial variation in encounter probability for each category, and $\varepsilon_{p} \left( {s_{i} c_{i} t_{i} } \right)$ represents the spatiotemporal variation in each category. Positive catch rates are modeled almost identically but with a log-linked predictor:

5 $$p_{2} \left( i \right) = \log \left[ {r\left( {s_{i} ,c_{i} ,t_{i} } \right)} \right] = \gamma_{r} \left( {c_{i} t_{i} } \right) + \omega_{r} \left( {s_{i} c_{i} } \right) + \varepsilon_{r} \left( {s_{i} c_{i} t_{i} } \right)$$

where the three parameters estimate the temporal, spatial, and spatiotemporal variability respectively, for each category for the positive catch rates. For both linear predictors, the temporal intercepts and spatial parameters were treated as fixed effects, and the spatiotemporal parameters were treated as autoregressive random effects. Model convergence was evaluated by ensuring that the gradient of the approximated marginal log-likelihood for all fixed effects was < 10^–6^ and that the Hessian matrix was positive definite at the maximum-likelihood estimates.

We used model-based estimates of the effective area occupied and centre of gravity to identify changes in the range and location of flatfish distributions^26^. Centre of gravity (longitude and latitude) was estimated by

6 $$\overline{x}\left( {c,t} \right) = \frac{{\mathop \sum \nolimits_{s = 1}^{{n_{s} }} d\left( {s,c,t} \right) \times x\left( s \right)}}{{\mathop \sum \nolimits_{i = 1}^{{n_{s} }} d\left( {s,c,t} \right)}}$$

where $x\left( s \right)$ is a latitudinal or longitudinal description of location for knot *s* and $d\left( {s,c,t} \right)$ is the predicted density across knots, categories, and time.

Effective area occupied ($h_{t}$) was estimated by

7 $$h_{t} = \frac{{b_{t} }}{{m_{t} }} = \frac{{\left( {\smallint D_{t} \left( s \right)ds} \right)^{2} }}{{\smallint D_{t}^{2} \left( s \right)ds}}$$

where $b_{t}$ is total abundance, $m_{t}$ is average population density (kg km^−2^), and $D_{t} \left( s \right)$ is the density function for that year. The estimation of this metric is done within VAST and Eq. (7) is a simplification used for brevity (for a full derivation, description, and validation see ref.^23^). This formulation of effective area occupied measures the area required to contain a population given its average population density. As a result, this metric can identify changes in area occupied regardless of changes in total abundance. To identify potential density-dependent changes in distribution, we used Pearson correlation to relate the estimated total biomass index with the effective area occupied for both species.

### Testing for density-dependent habitat selection

We wanted to account for the effects of density-dependent habitat selection to ensure that distribution shifts were not the result of changes in biomass. We tested for the existence of density-dependent habitat selection by examining the relationship between local and global biomass^28,66^. This relationship serves as a proxy for habitat suitability because theory predicts that when density-dependent habitat selection exists, increased abundance will decrease population growth rates in optimal habitats and increase growth rates in marginal habitats^27^. We considered the null hypothesis to be that variation in local density responses is independent of habitat suitability (i.e. a single positive linear or exponential relationship between local and total population biomass). This relationship would indicate that as global biomass increases, so does density at all locations. Using the output of the VAST, we used a simple linear regression to model the relationship between annual global biomass estimates and annual local density estimates (at all 50 knots). If there was a positive relationship and no evidence of model misfit (e.g. non-normal residuals would indicate locally varying relationships) this would serve as evidence for the null hypothesis (density-dependent habitat selection does not play a large role in local population growth rates). If there was no relationship and/or evidence of model misfit, this would serve as evidence for the alternative hypothesis and we would test whether there are location specific relationships between local density and global biomass.

If there was evidence against the null hypothesis, we wanted to account for the effects of density-dependent habitat selection. To accomplish this, we developed a non-linear random effects model using,

8 $$\widehat{{y_{k} }} = a_{k} x^{{b_{k} }}$$

to examine local variability in the density-dependent habitat selection relationship^28,66,67^. Where $\widehat{{y_{k} }}$ is the log of local density + 10, with the + 10 ensuring that all $y_{k}$’s are positive (the log of local density was < 1 for some knots in some years), and x representing the log of total biomass + 10. Both $a_{k}$ and $b_{k}$ are random effects to provide unique estimates for all knots. We would expect that the estimate of $b_{k}$ for locations would be least/concave in prime/core habitats and highest/convex in marginal habitats. Weaker responses (i.e. $b_{k}$ ≤ 1) would specifically indicate that a location is not sensitive to regional biomass changes. Since the residuals should represent deviations from the density-dependent relationship (i.e. the density-independent influence on distribution) we used them in the remaining analyses to ensure that our results would not be affected by density-dependent habitat selection. The model was fit in Template Model Builder (TMB)^68^ in R^69^, where convergence was assessed using the same criteria that we used for the VAST model.

### Correlation analysis for recovery response

Knot specific density estimates (or residuals) were correlated with knot specific bottom-water temperature estimates across all years using rank-based Spearman correlation,

9 $$d_{{s,t_{i} }} = \frac{{cov\left( {rnk\left( {d_{{s,t_{i} }} } \right), rnk\left( {temp_{{s,t_{j} }} } \right)} \right)}}{{\sigma_{{rnk\left( {d_{{s,t_{i} }} } \right)}} \sigma_{{rnk\left( {temp_{{s,t_{j} }} } \right)}} }}$$

Rank correlation allowed a comparison of the relative densities and temperatures, such that total population size and warmer or colder years would not affect correlations. Since both species modified their distributions in unique ways and magnitudes, we opted to use relative correlation between years rather than identifying a threshold of correlation as a cut-off to decide which years were correlated. In addition, we correlated the spatial distribution of temperature and fish with themselves across time (Figs. S6, S18, and S19) to identify their relative change in regards to their historical distributions.

## Supplementary Information

Supplementary Information

## Data Availability

The data analyzed during the current study are available from Fisheries and Oceans Canada at The Northwest Atlantic Fisheries Centre but restrictions apply to the availability of these data, which were used due to collaboration with Fisheries and Oceans Canada scientists, and are not publicly available. Data are however available from the authors associated with Fisheries and Oceans Canada and with permission of Fisheries and Oceans Canada.
