# Supplementary material for: Lagged recovery of fish spatial distributions following a cold-water perturbation
Source: Sci Rep. 2021 May 4;11:9513. doi: 10.1038/s41598-021-89066-x (PMC8096816; doi:10.1038/s41598-021-89066-x)
Supplement: Supplementary file 1 — Supplementary Information [file 41598_2021_89066_MOESM1_ESM.docx]

**Supporting Information (SI Appendix)**

**
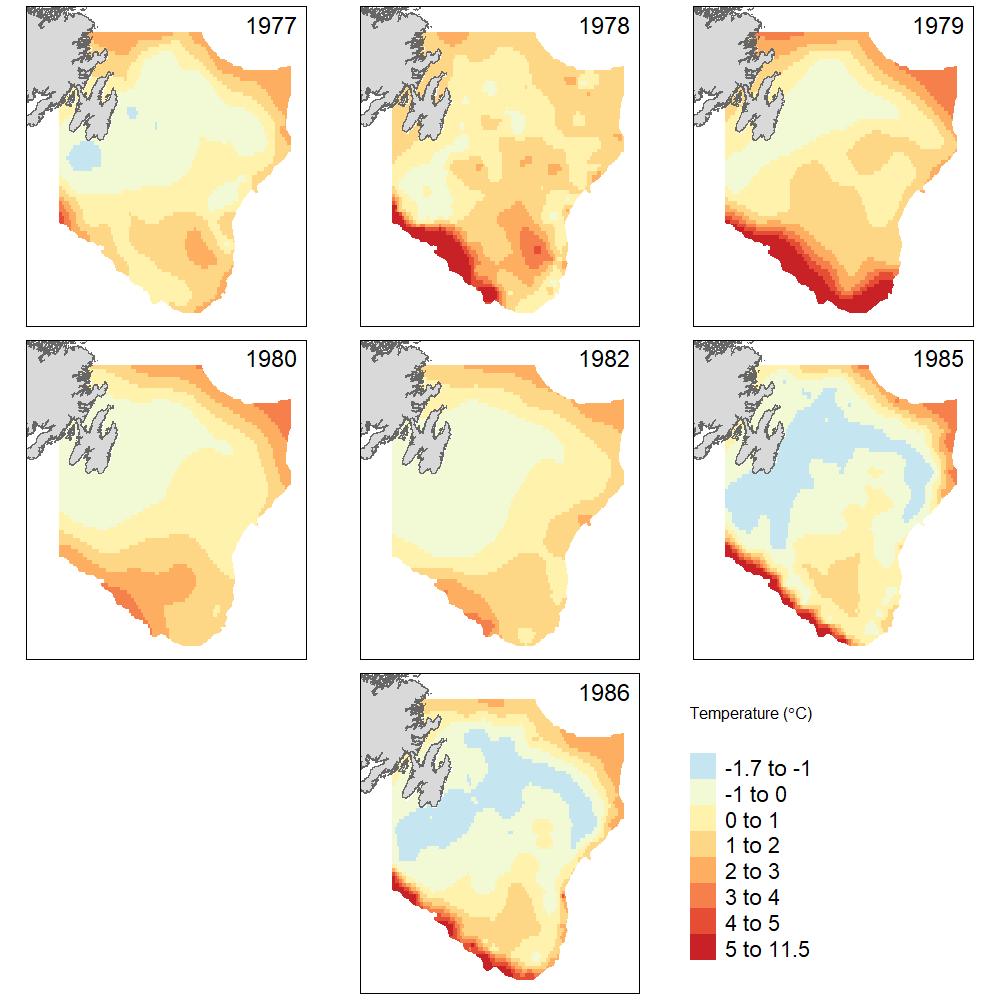
**

Figure 1. Kriged, non-modified, survey temperatures from 1977-1986, 100x100 raster grid. Darkest red represents all temperatures above 5^°^ C because those temperatures have a very limited spatial coverage. Figure made using R (version 3.6.2. https://www.r-project.org/)^1^ and the packages sp^2^, raster^3^, rgeos^4^, and RColorBrewer^5^. Shapefiles for land and bathymetry were obtained from Natural Earth (https://www.naturalearthdata.com/downloads/10m-physical-vectors/10m-bathymetry/).


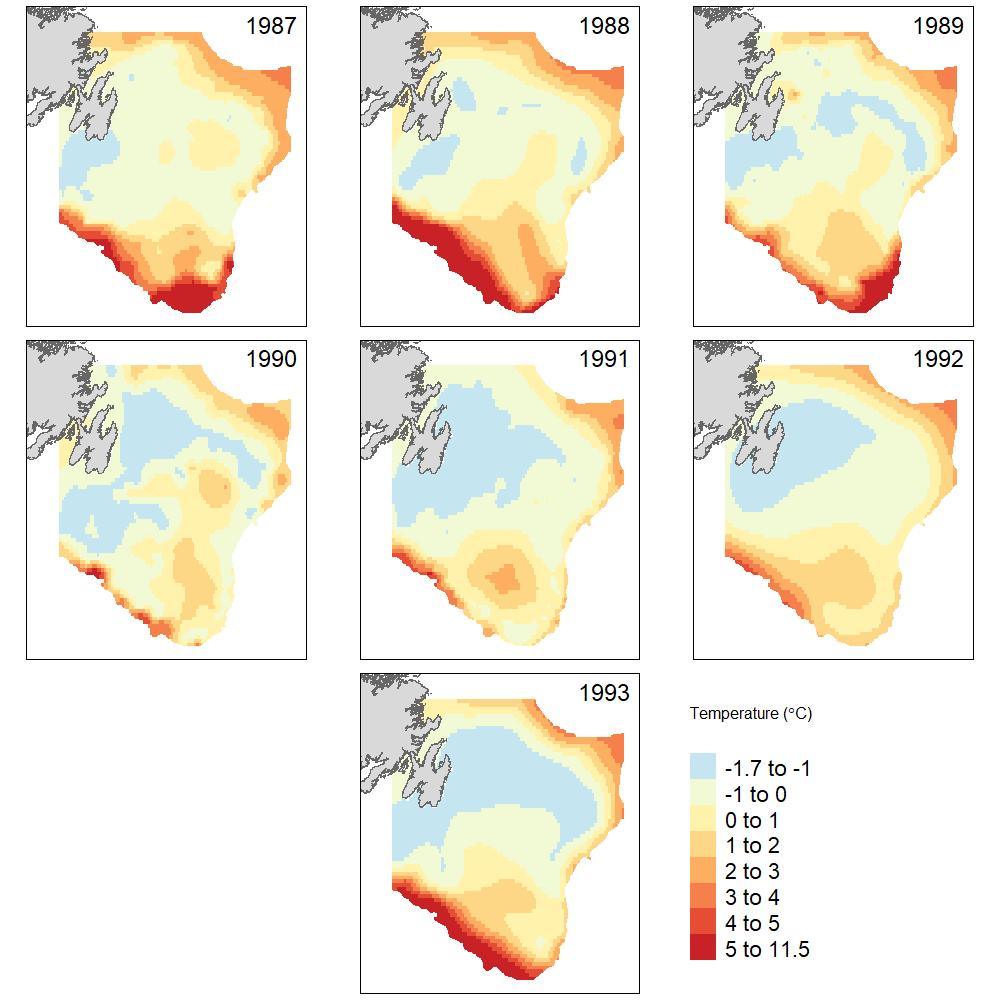


Figure 2. Kriged, non-modified, survey temperatures from 1987-1993, 100x100 raster grid. Darkest red represents all temperatures above 5^°^ C because those temperatures have a very limited spatial coverage. Figure made using the software described in Figure 1.


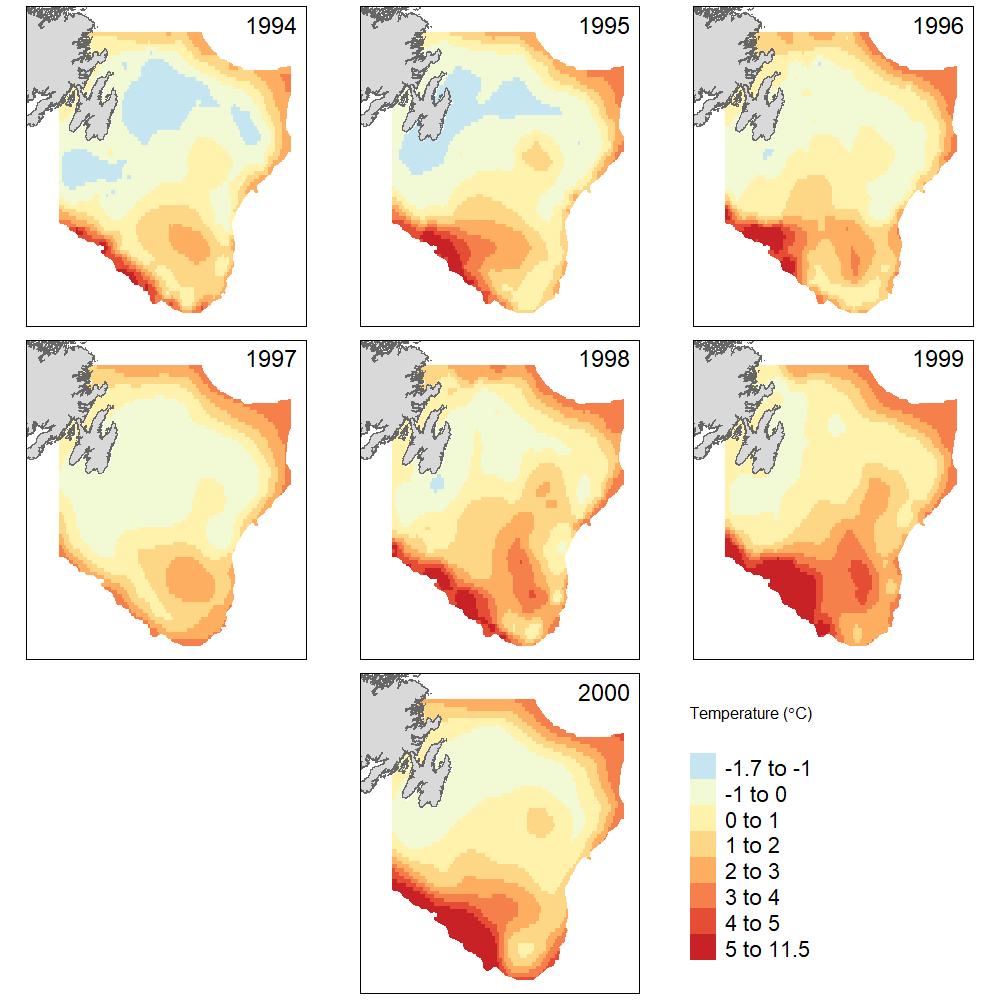


Figure 3. Kriged, non-modified, survey temperatures from 1994-2000, 100x100 raster grid. Darkest red represents all temperatures above 5^°^ C because those temperatures have a very limited spatial coverage. Figure made using the software described in Figure 1.


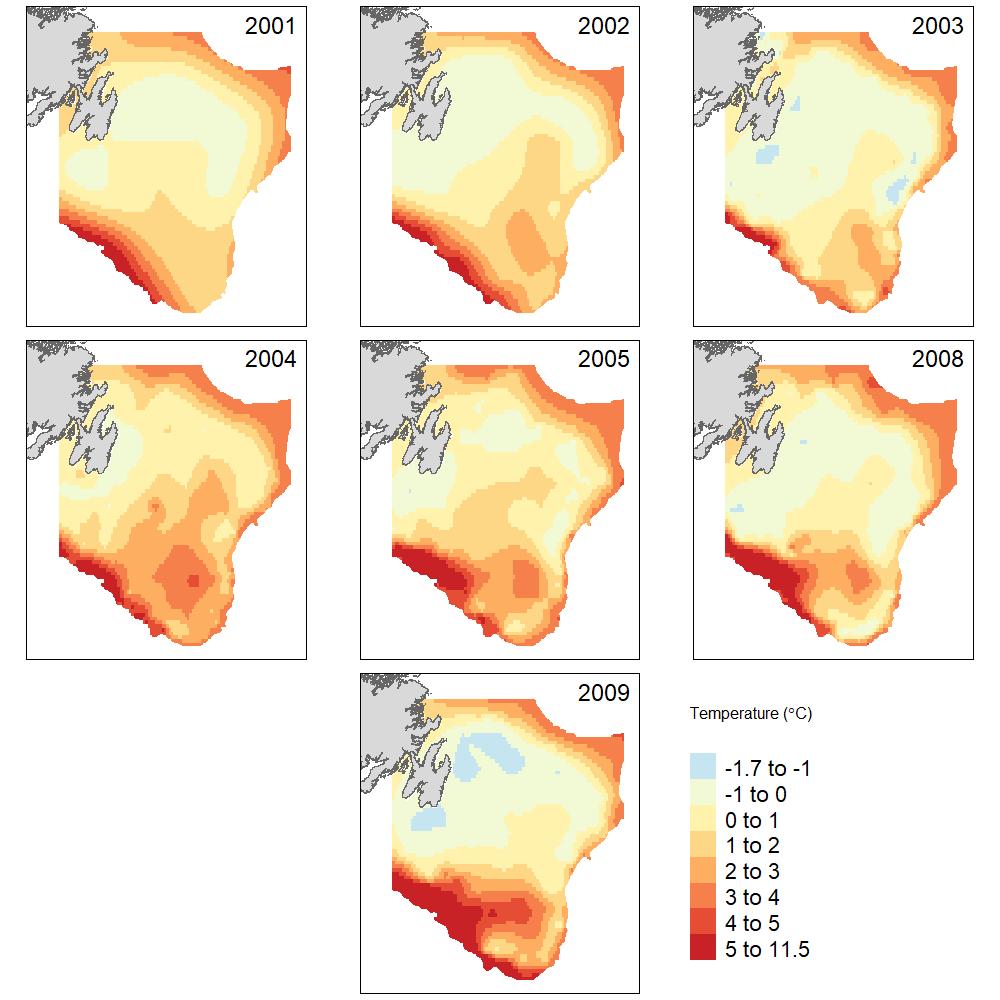


Figure 4. Kriged, non-modified, survey temperatures from 2001-2009, 100x100 raster grid. Darkest red represents all temperatures above 5^°^ C because those temperatures have a very limited spatial coverage. Figure made using the software described in Figure 1.


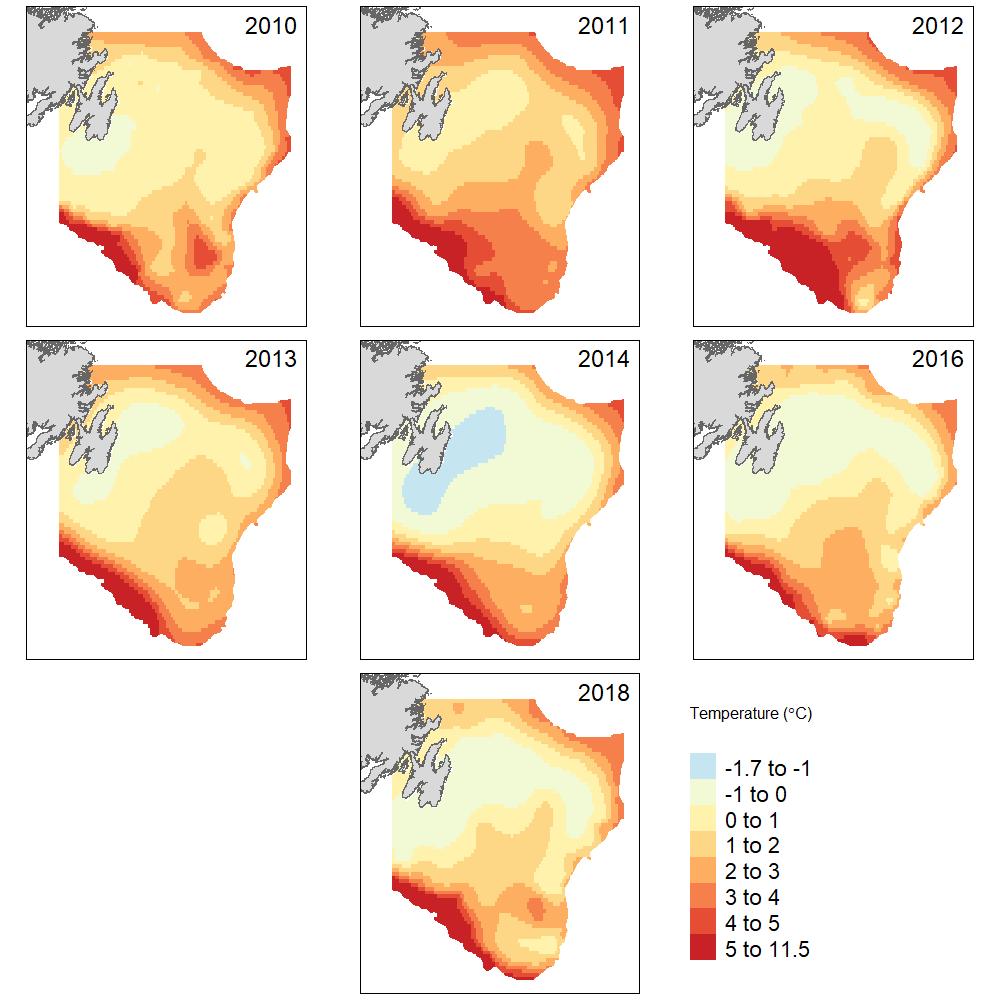


Figure 5. Kriged, non-modified, survey temperatures from 2010-2018, 100x100 raster grid. Darkest red represents all temperatures above 5^°^ C because those temperatures have a very limited spatial coverage. Figure made using the software described in Figure 1.


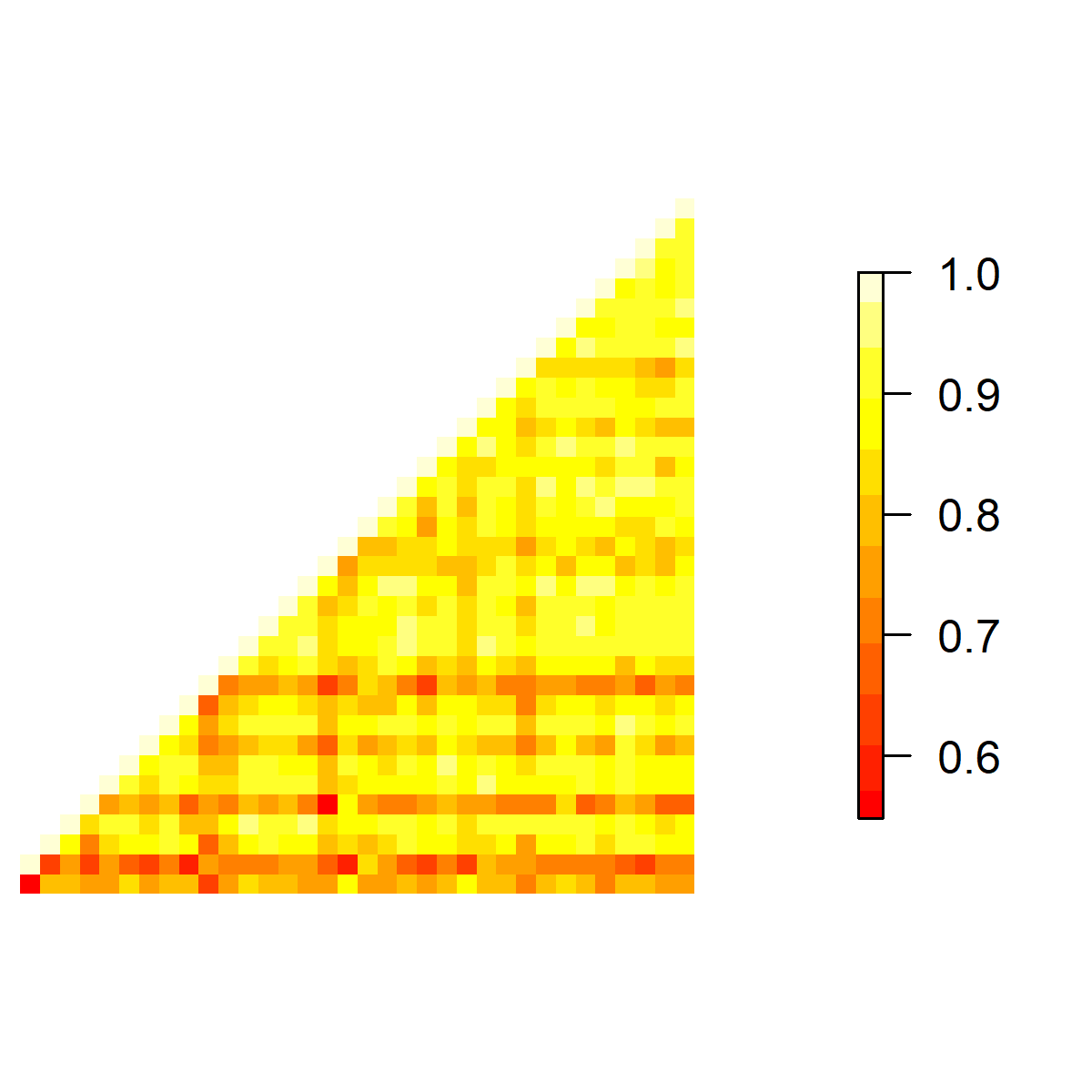


Figure 6. Spearman correlation of spatial temperatures over time (1977 – 2018) with itself. 1977 is on the left of the x-axis and bottom of the y-axis. Figure made using R (version 3.6.2. https://www.r-project.org/)^1^ and the packages raster^3^, fields^6^, and RColorBrewer^5^.


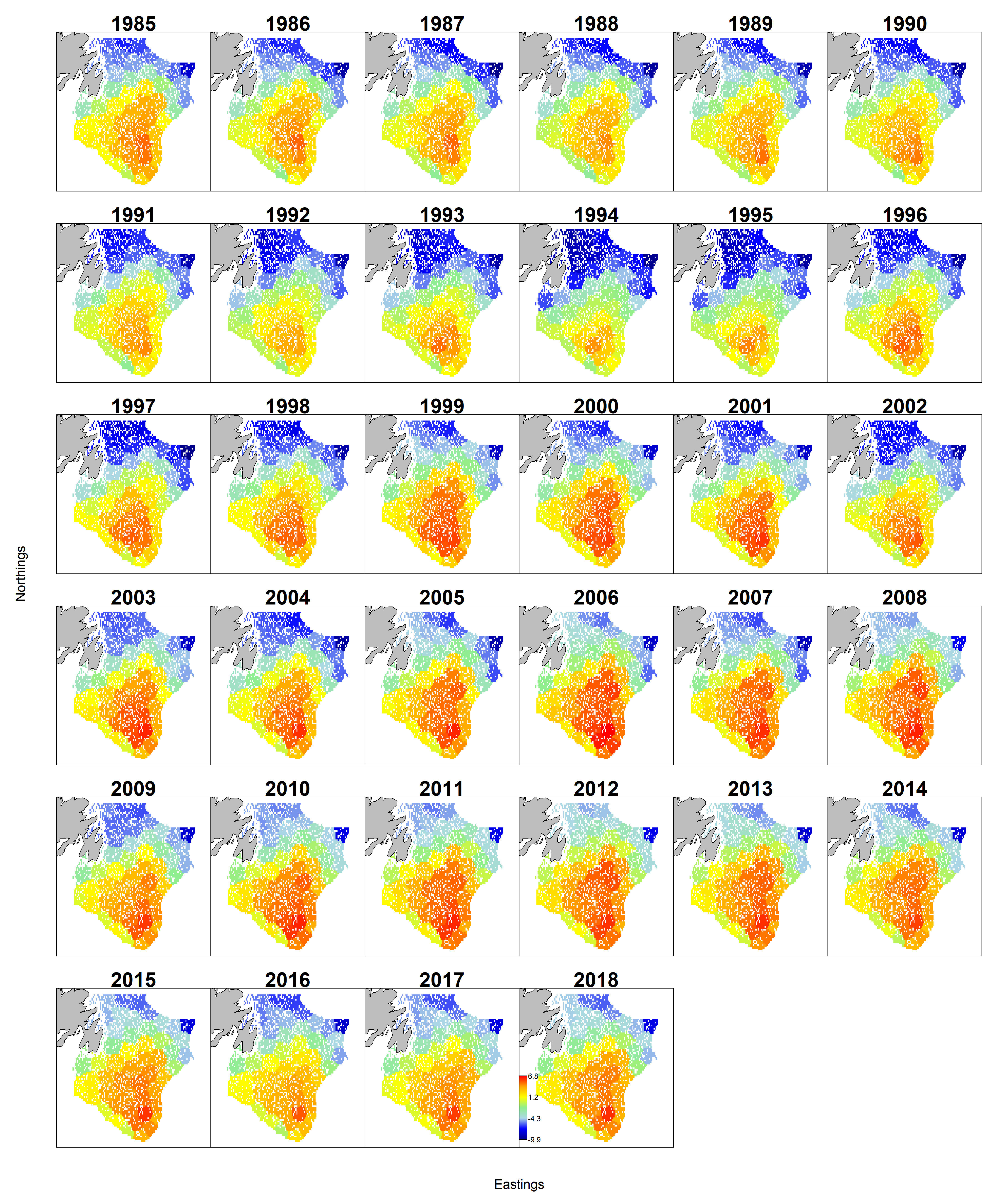


Figure 7. Yellowtail flounder distribution over time using the 50 knot VAST model, red indicates high density and blue indicates low density. Figure made using R (version 3.6.2. https://www.r-project.org/)^1^ and the package VAST^7^.


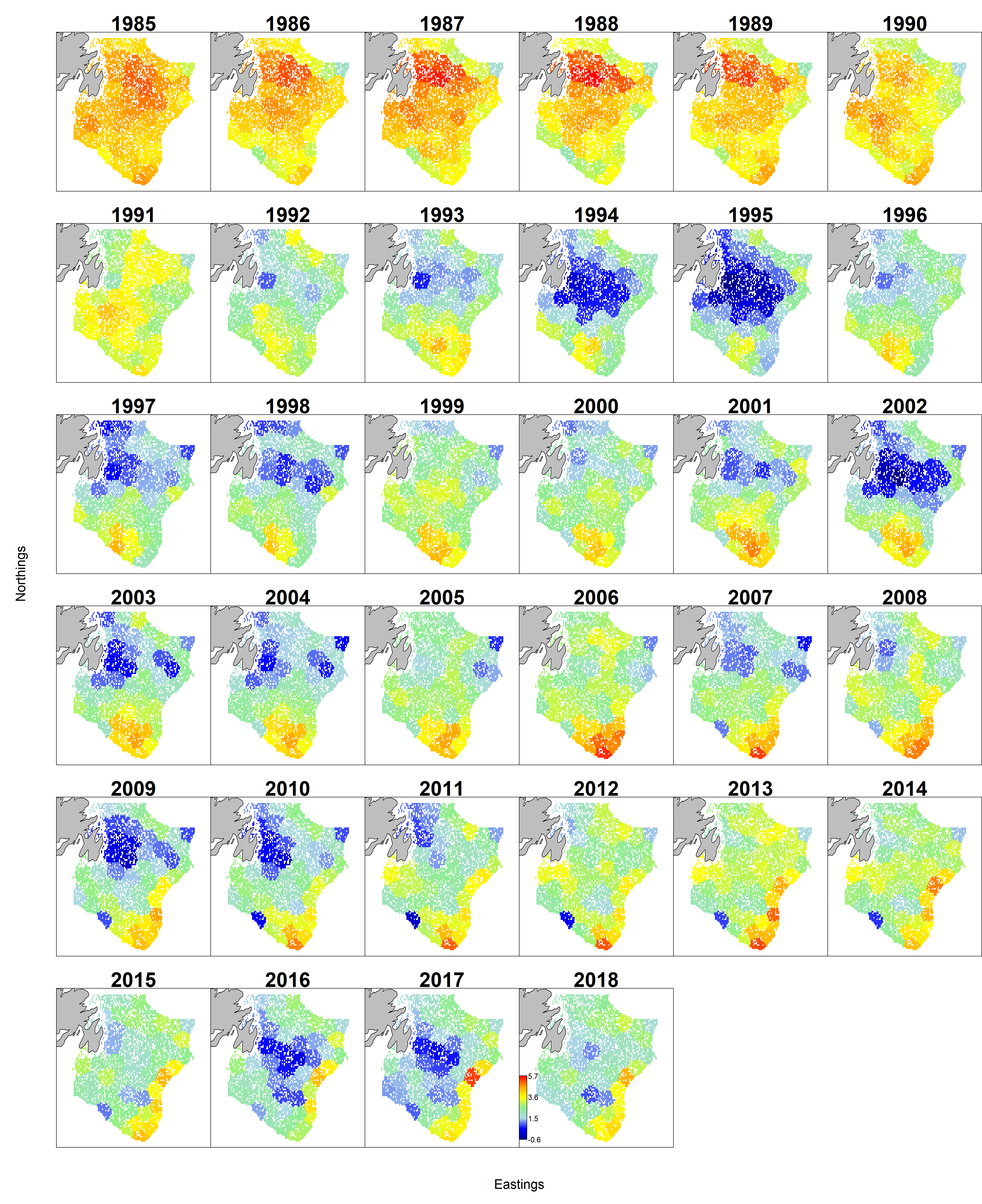


Figure 8. American plaice distribution over time using the 50 knot VAST model, red indicates high density and blue indicates low density. Figure made using the software described in Figure 7.


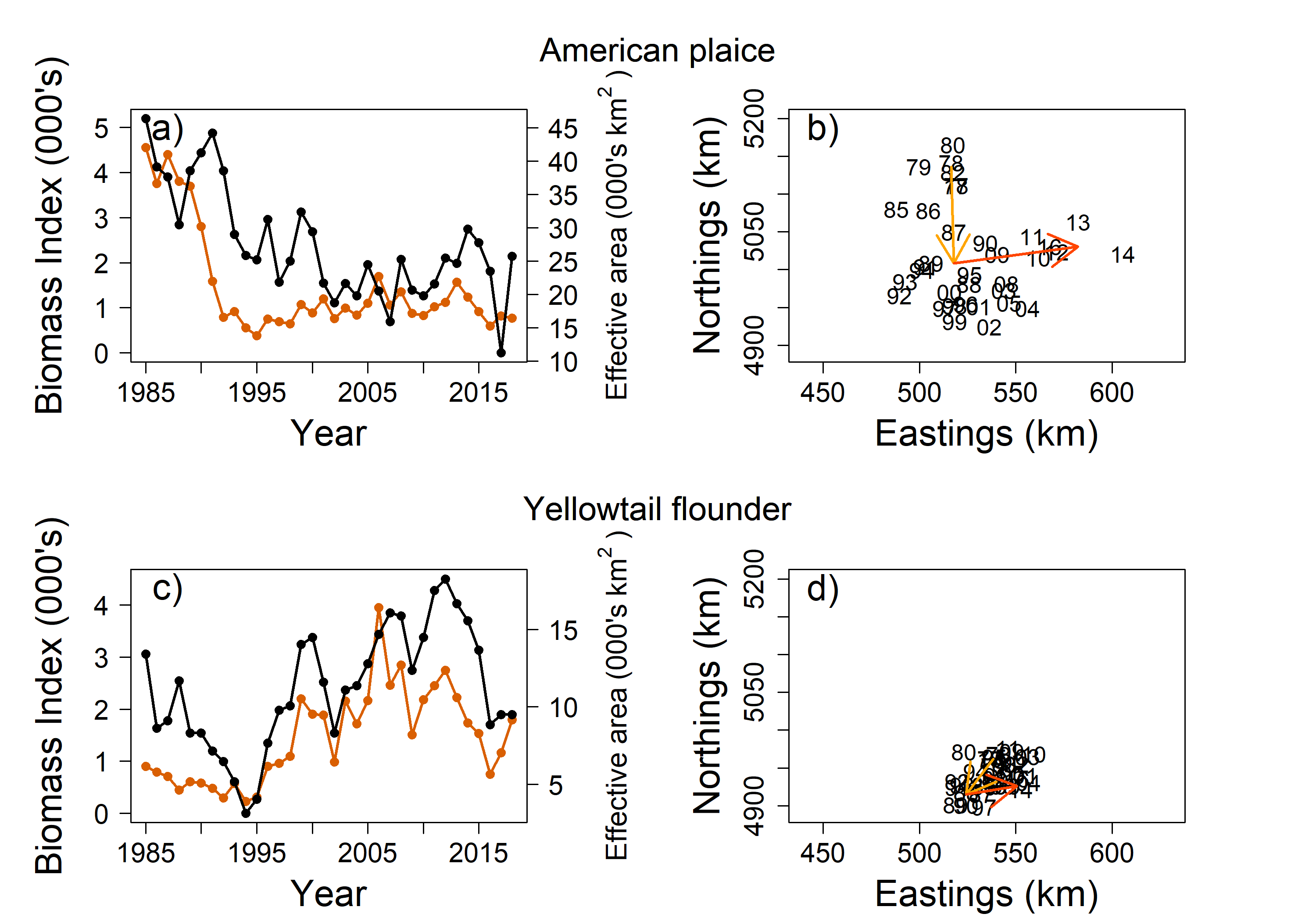


Figure 9. Biomass index and effective area for a) American plaice and c) yellowtail flounder (biomass index = orange lines, effective area = black lines). Center of gravity for b) American plaice (black text) and d) yellowtail flounder (black text). The orange arrows in b & d) represent the median direction of change of fish distributions from 1985 – 1993, and the red arrows represent the median change from 1993 – 2018. Figure made using R (version 3.6.2. https://www.r-project.org/)^1^.


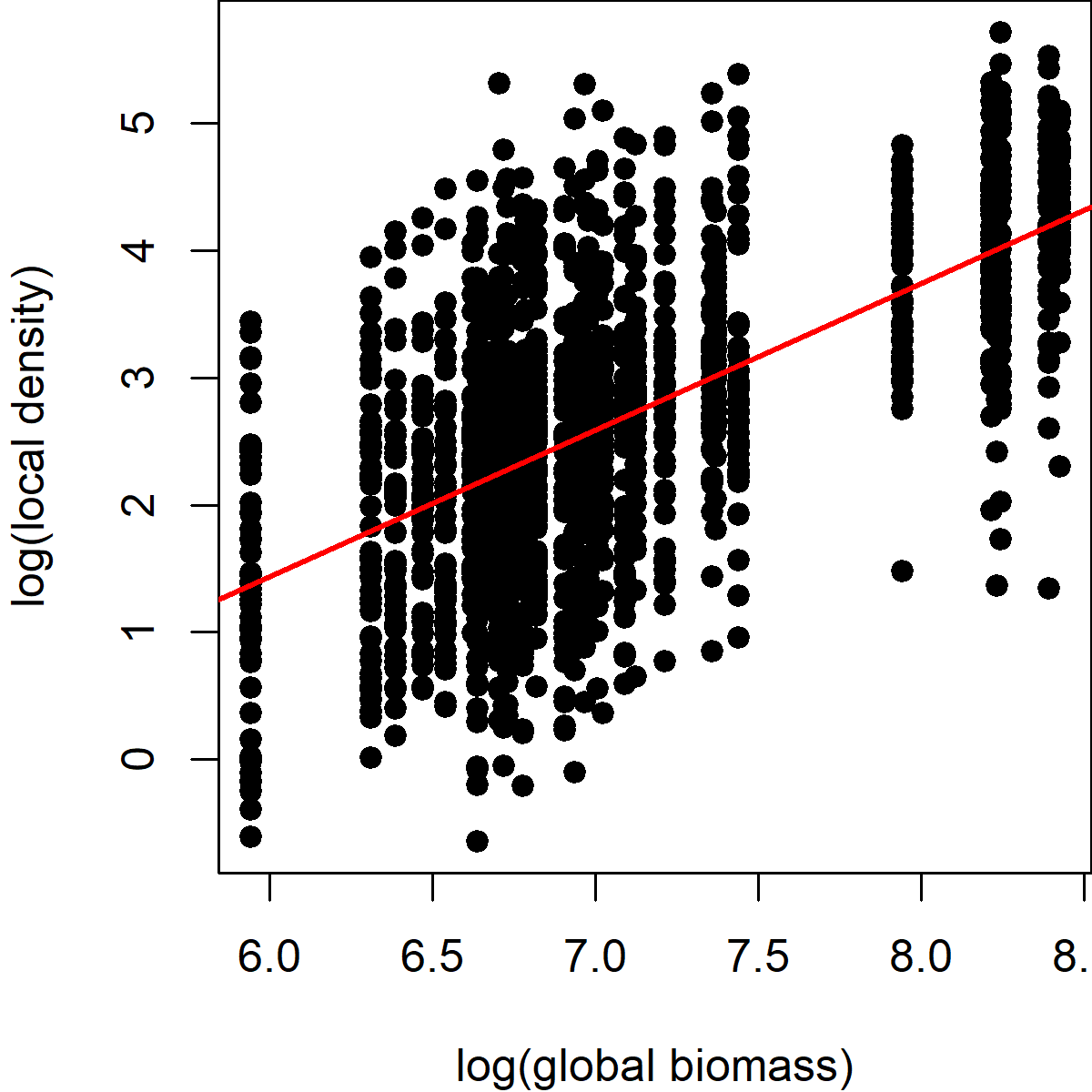


Figure 10. American plaice relationship between global (total) population biomass and local population density. The red line indicates the fit of a linear model. Figure made using R (version 3.6.2. https://www.r-project.org/)^1^.


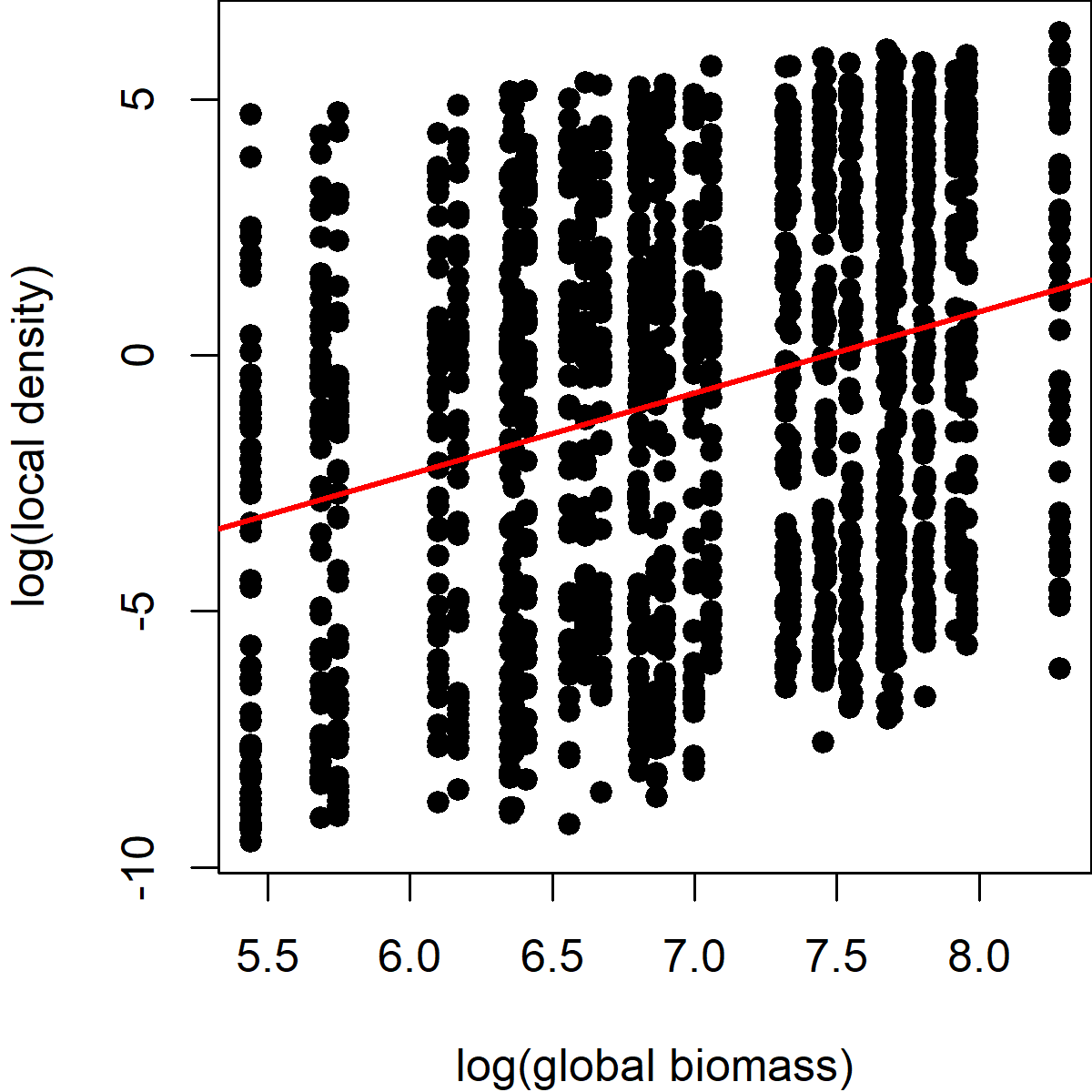


Figure 11. Yellowtail flounder relationship between global (total) population biomass and local population density. The red line indicates the fit of a linear model. Figure made using the same software as Figure 10.


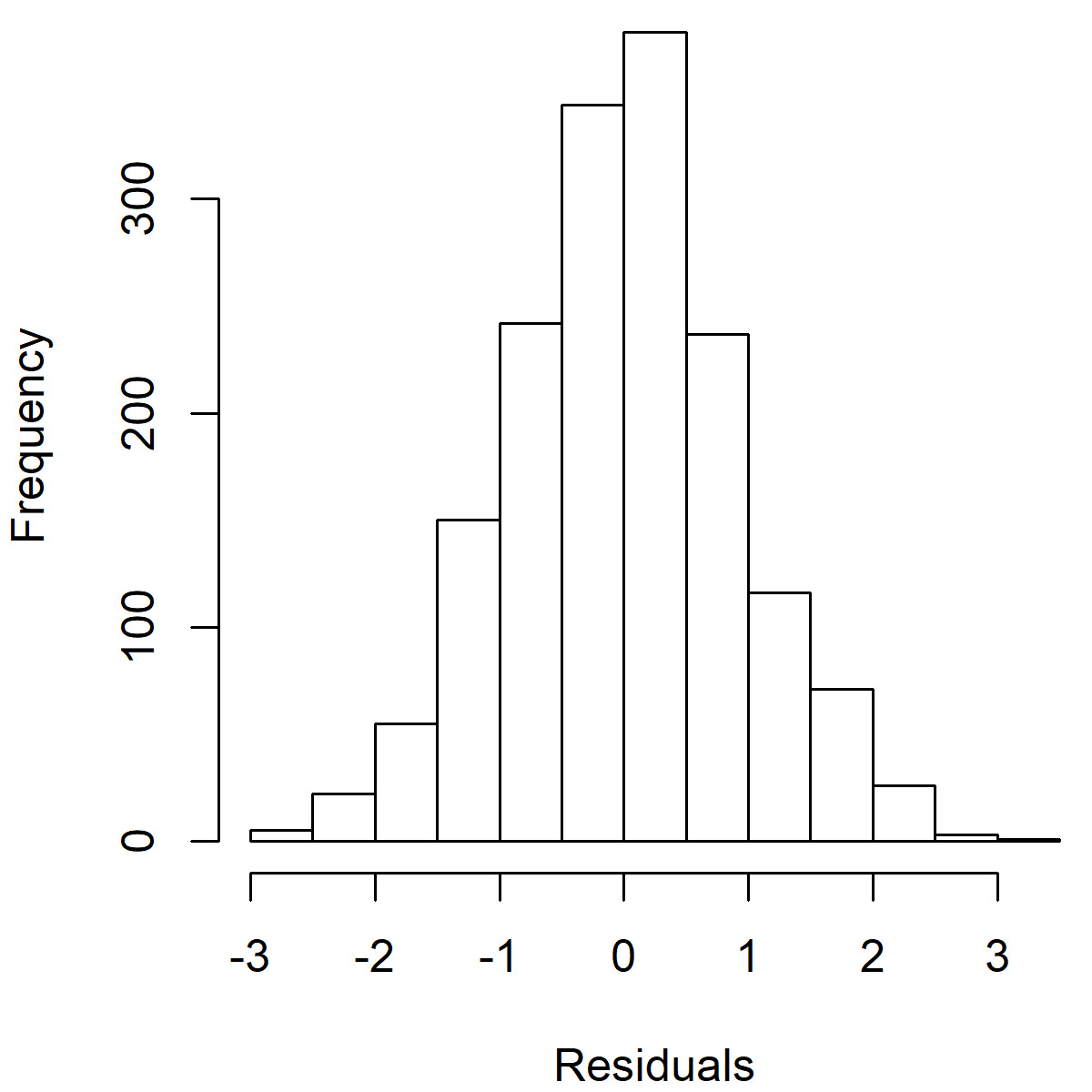


Figure 12. Histogram of residuals from the linear model between American plaice global population biomass and local population density. Figure made using the same software as Figure 10.


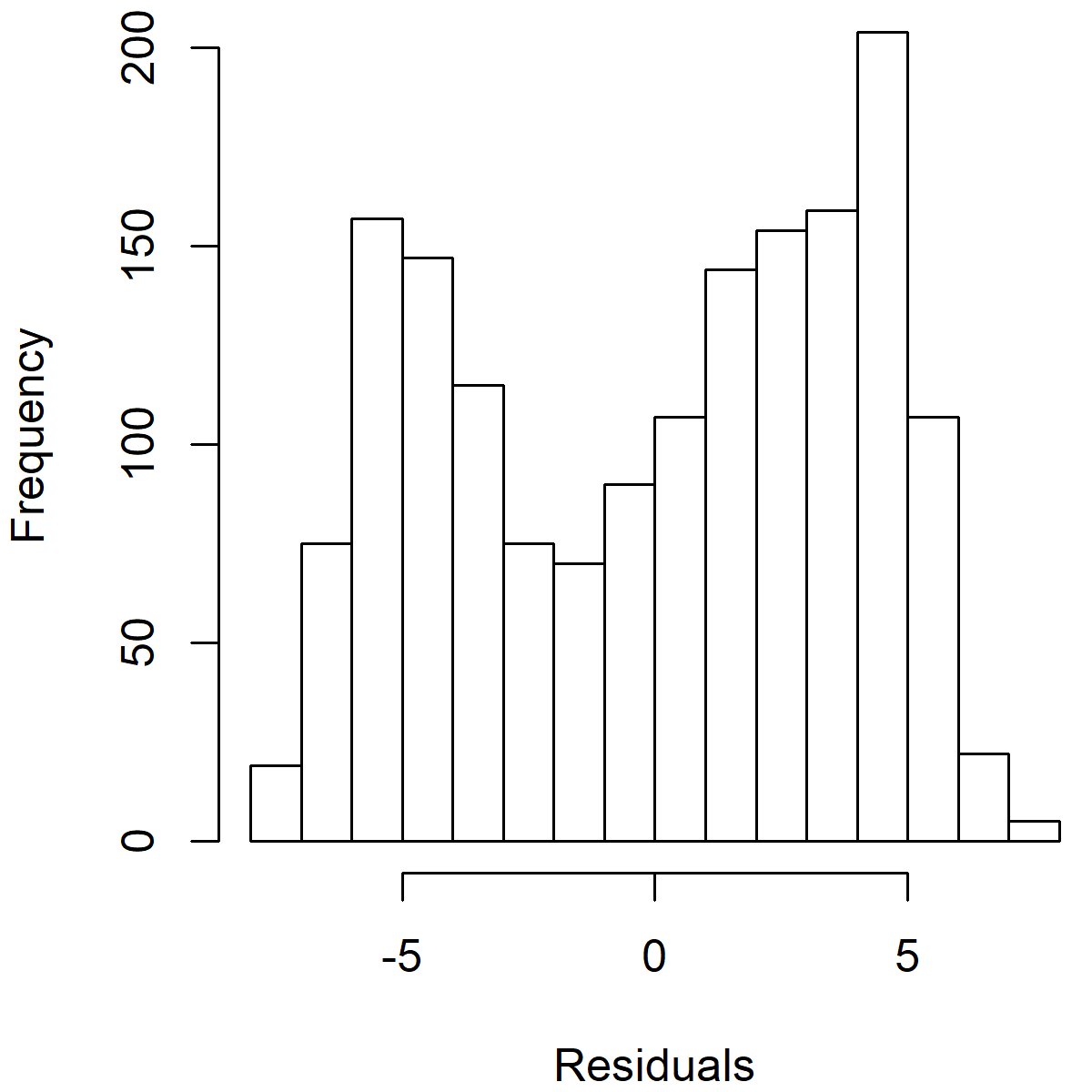


Figure 13. Histogram of residuals from the linear model between yellowtail flounder global population biomass and local population density. Figure made using the same software as Figure 10.


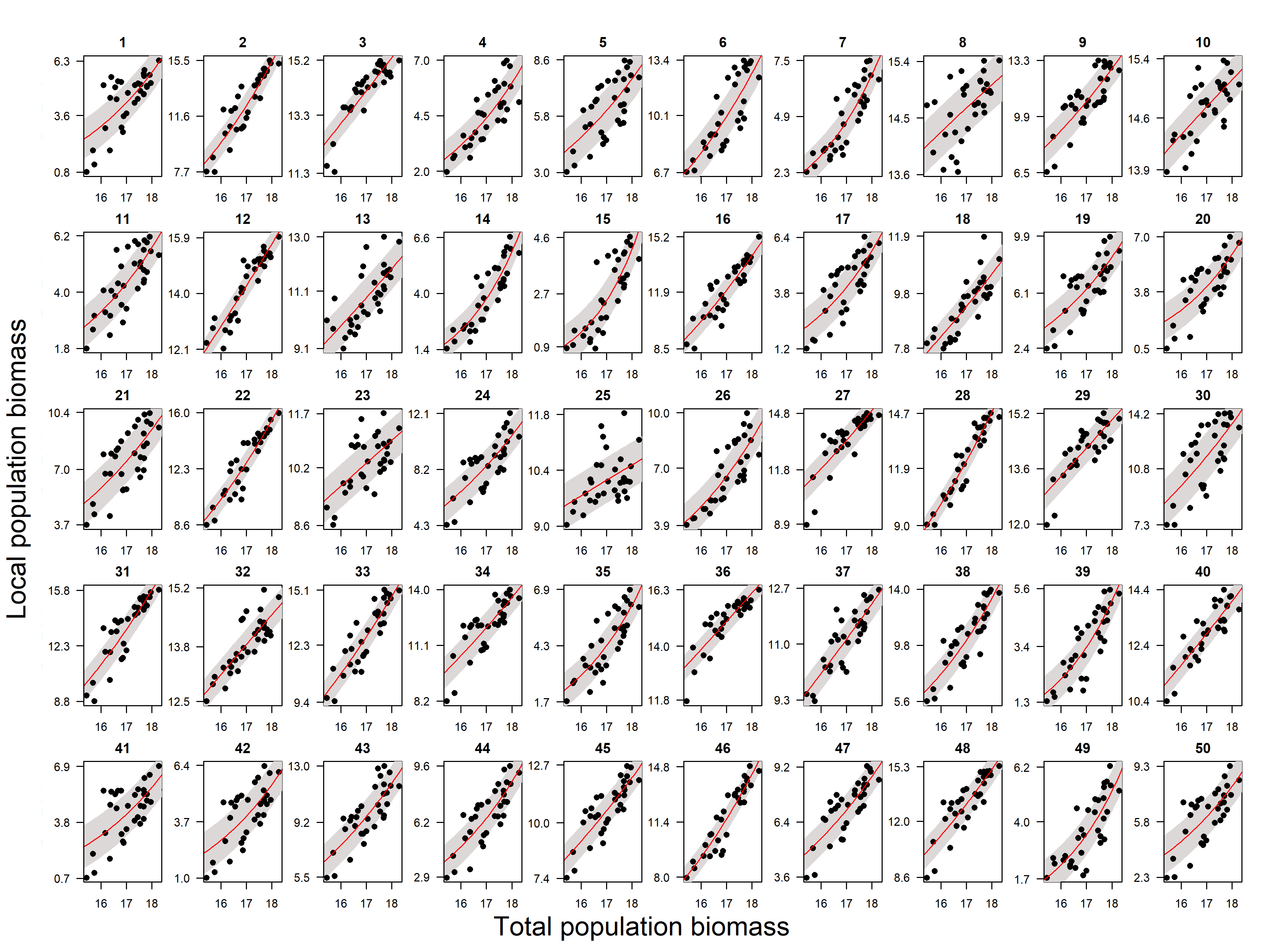


Figure 14. Yellowtail flounder relationships between global (total) population biomass (x-axis) and local population biomass (y-axis) at all 50 knots (panels) from the VAST. The red lines represent the predicted local relationship from the random effects model and grey polygon represents the root mean square error for those relationships. Figure made using R (version 3.6.2. https://www.r-project.org/)^1^ and the package ggplot2^8^.


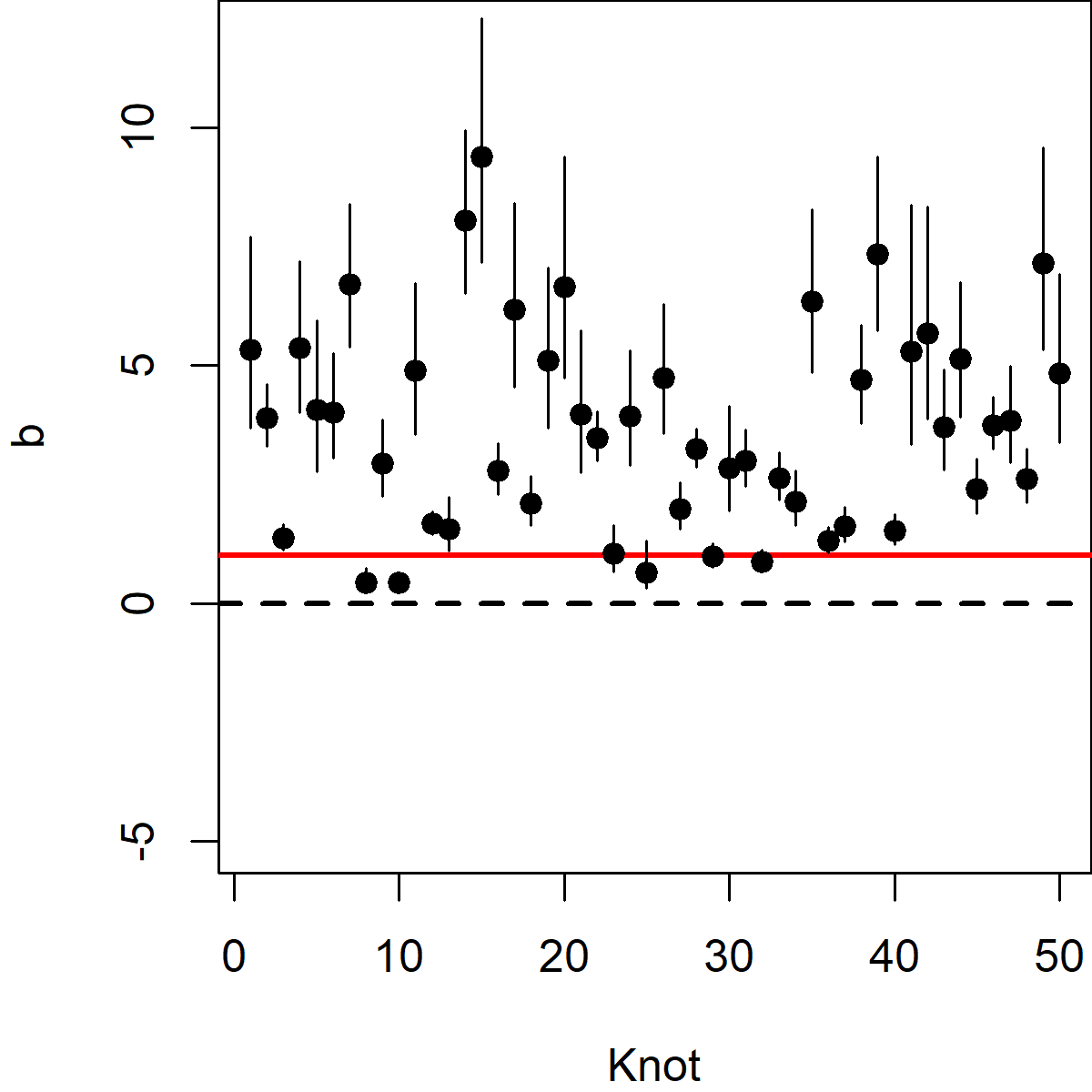


Figure 15. Model estimates of b_k_ at each knot. The segments represent +/- 1.96*SD. The solid red line indicates 1, the dashed black line indicates 0. Figure made using R (version 3.6.2. https://www.r-project.org/)^1^.


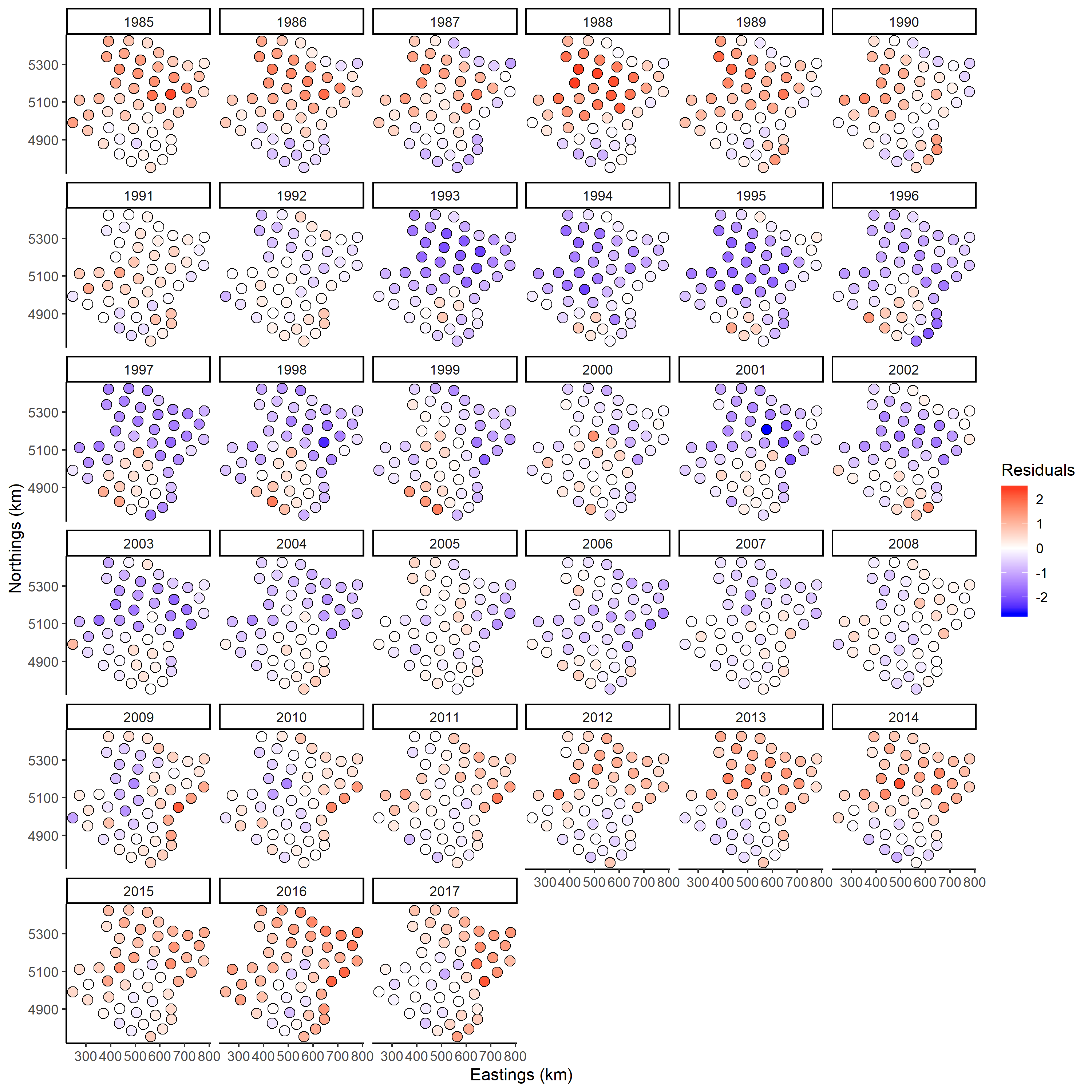


Figure 16. Yellowtail flounder local density residuals from the density dependent habitat selection model. Figure made using R (version 3.6.2. https://www.r-project.org/)^1^ and the packages sp^2^ and ggplot2^8^.


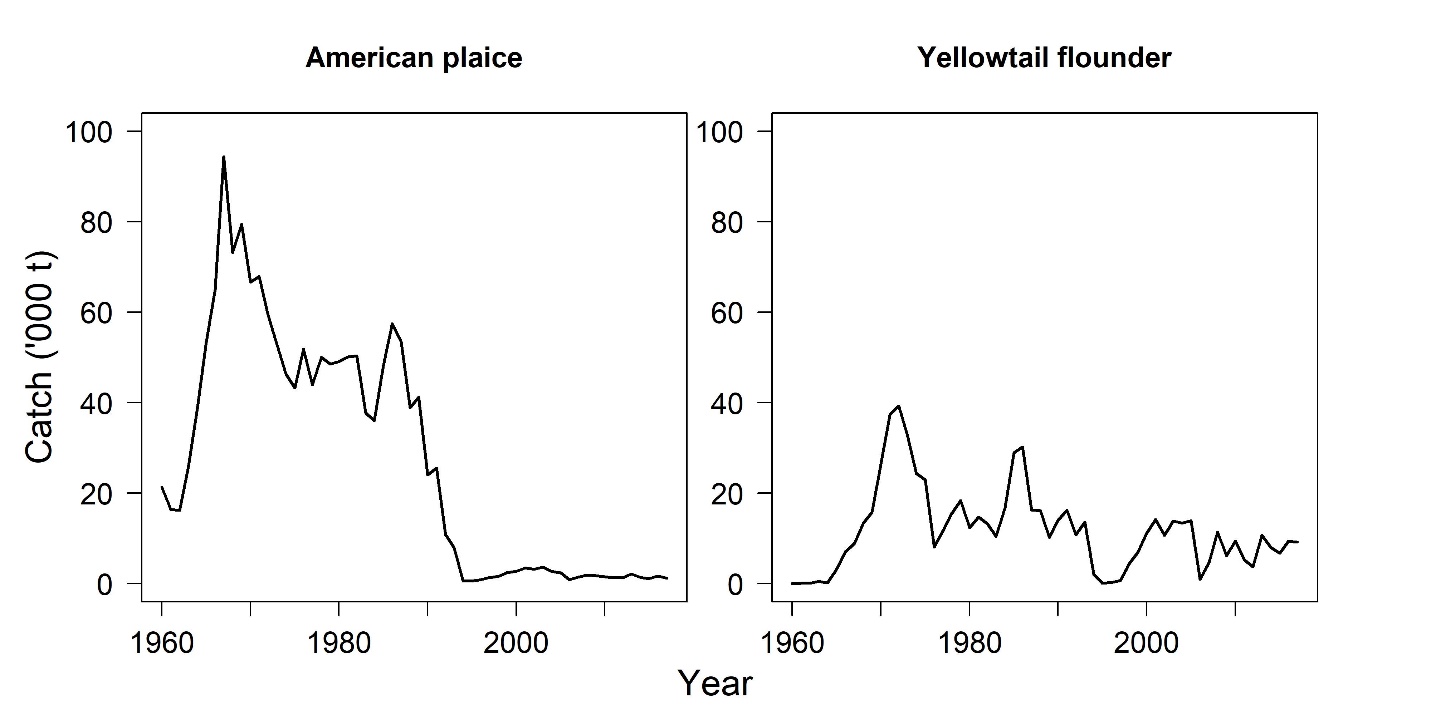


Figure 17. Time-series of catch (‘000 t) for American plaice^9^ and yellowtail flounder^10^ in NAFO divisions 3LNO. Figure made using R (version 3.6.2. https://www.r-project.org/)^1^.


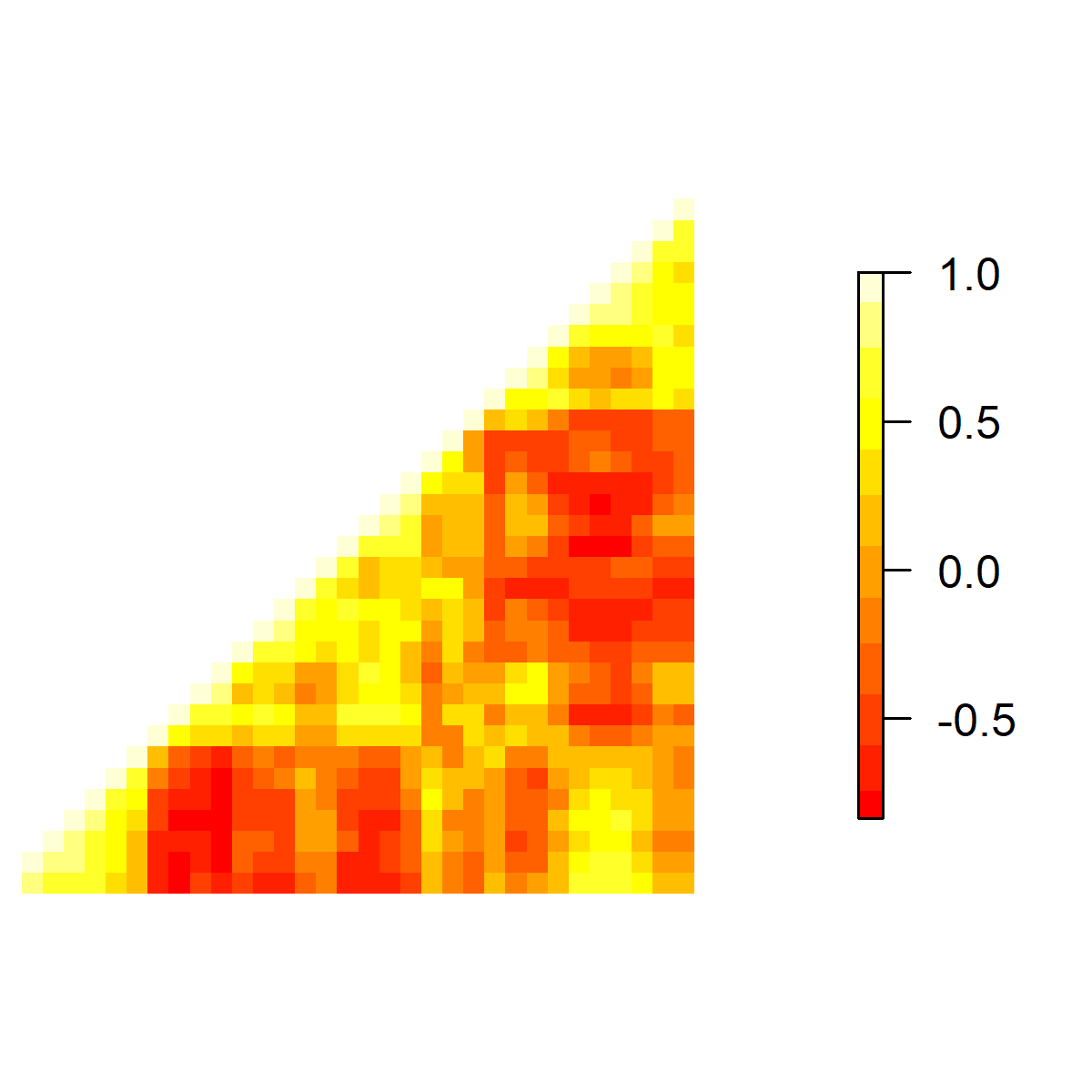


Figure 18. Spearman correlation of the spatial distribution of yellowtail flounder density-dependent residuals over time (1985 – 2018) with itself. 1985 is on the left of the x-axis and bottom of the y-axis. Figure made using the same software as Figure 6.


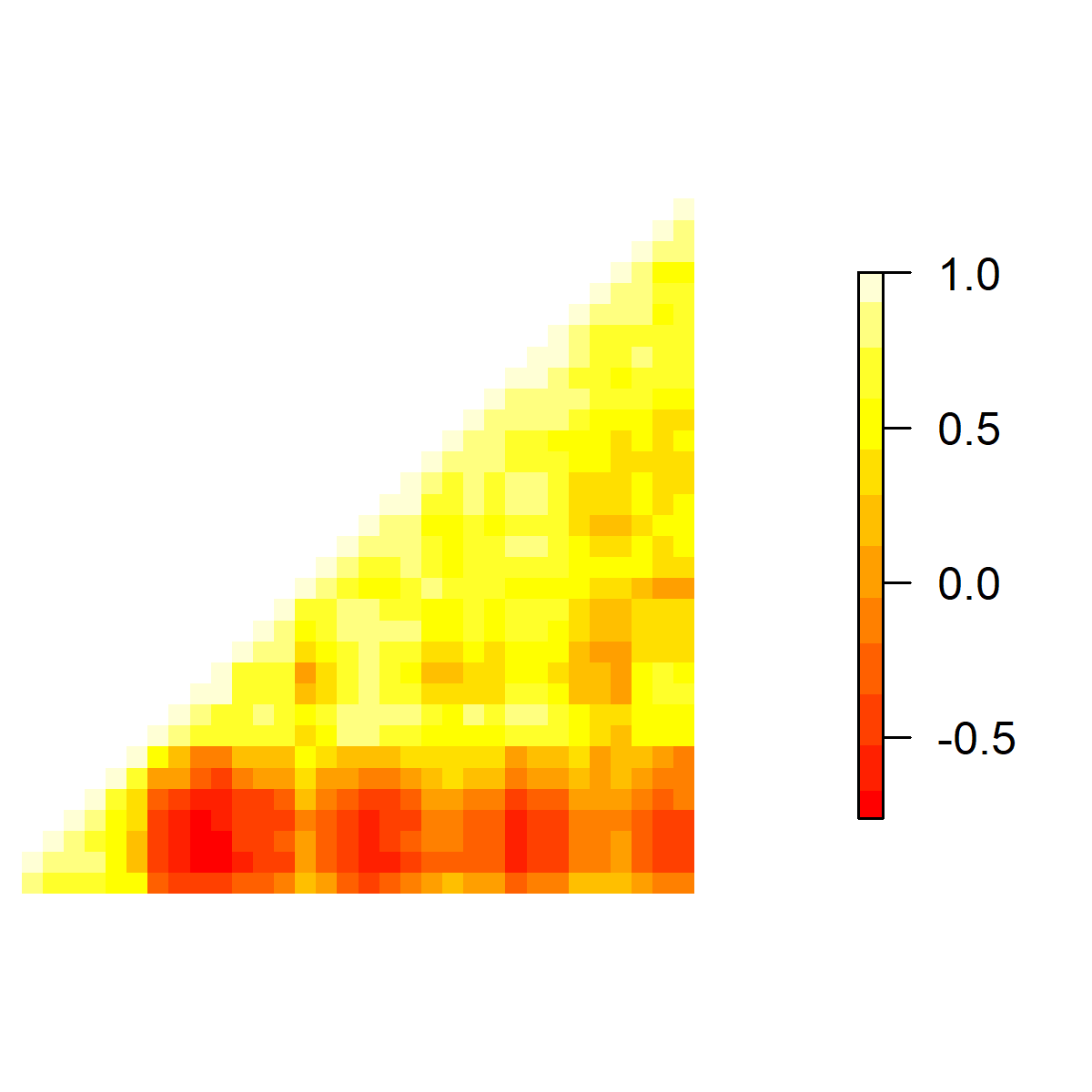


Figure 19. Spearman correlation of the spatial distribution of American plaice over time (1985 – 2018) with itself. 1985 is on the left of the x-axis and bottom of the y-axis. Figure made using the same software as Figure 6.


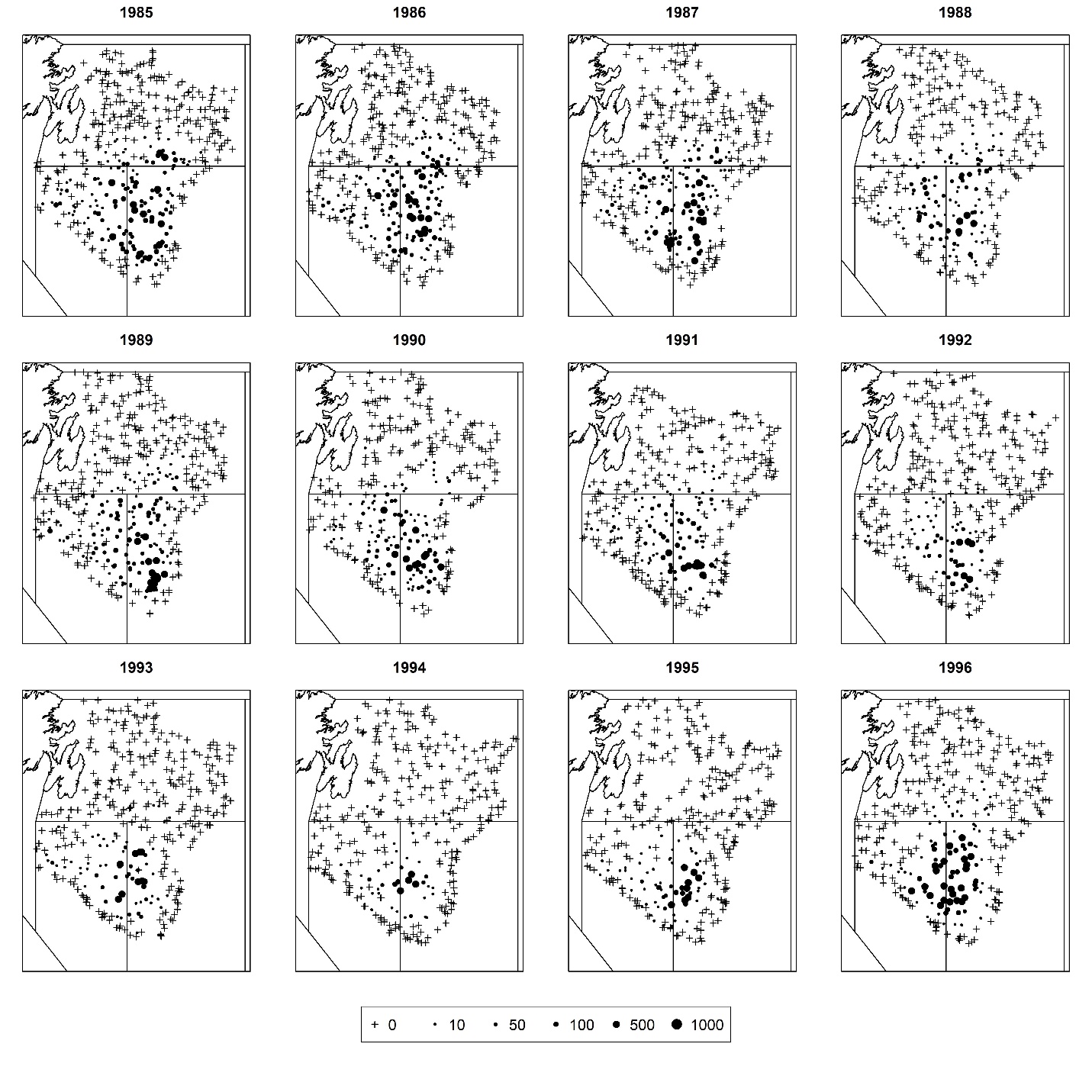


Figure 20. Observed survey catches for yellowtail flounder in NAFO divisions 3LNO from 1985 – 1996. No catch is denoted as a plus symbol and positive catches (biomass [kg]) are shown as points that increase in size with increased biomass caught. Figure made using R (version 3.6.2. https://www.r-project.org/)^1^ and the packages sp^2^, sf^11^, and rgdal^12^. Bathymetry shapefiles were taken from Natural Earth (<https://www.naturalearthdata.com/downloads/10m-physical-vectors/10m-bathymetry/>) and the NAFO division shapefile was taken from NAFO (https://www.nafo.int/Data/GIS).


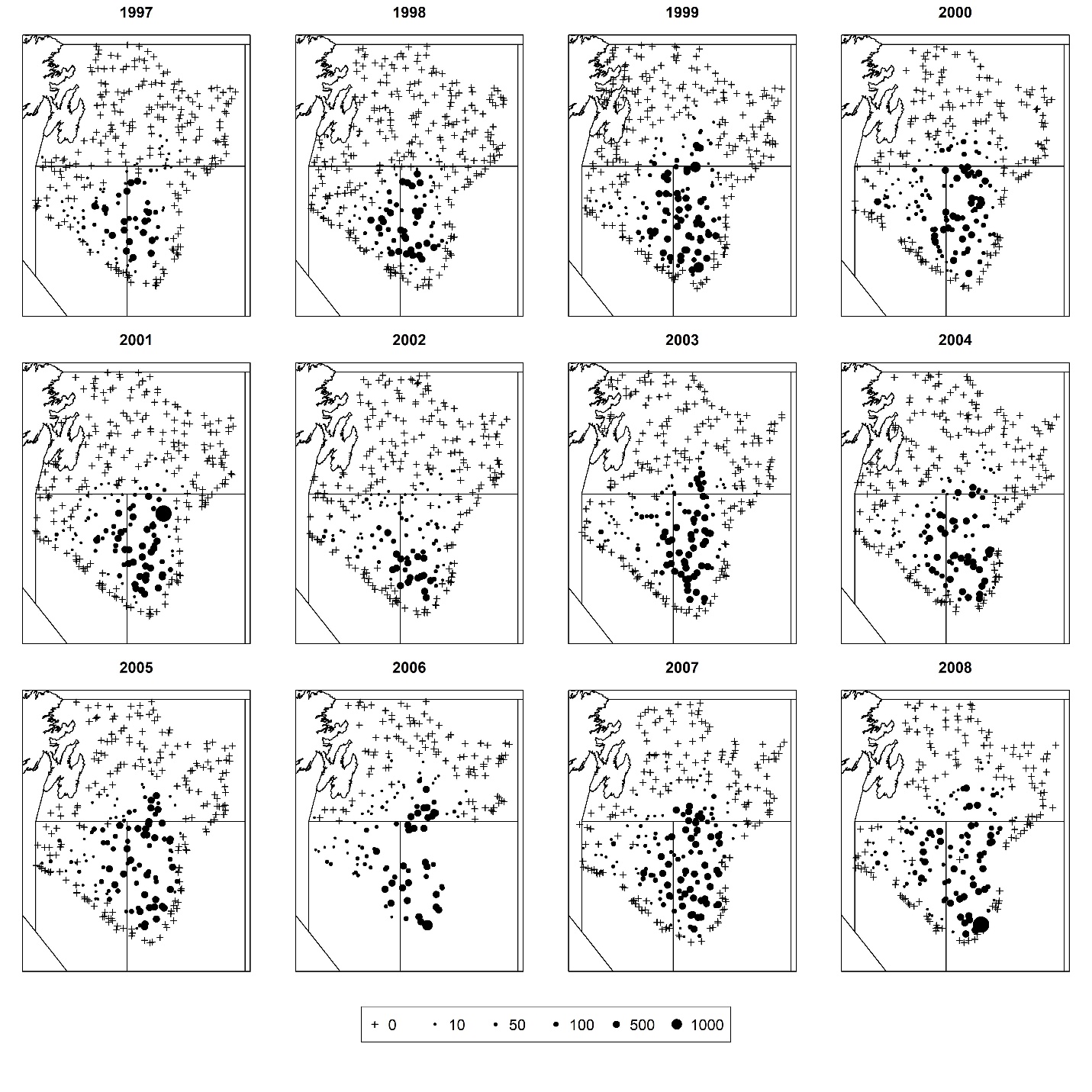


Figure 21. Observed survey catches for yellowtail flounder in NAFO divisions 3LNO from 1997 – 2008. No catch is denoted as a plus symbol and positive catches (biomass [kg]) are shown as points that increase in size with increased biomass caught. Figure made using the same software as Figure 20.


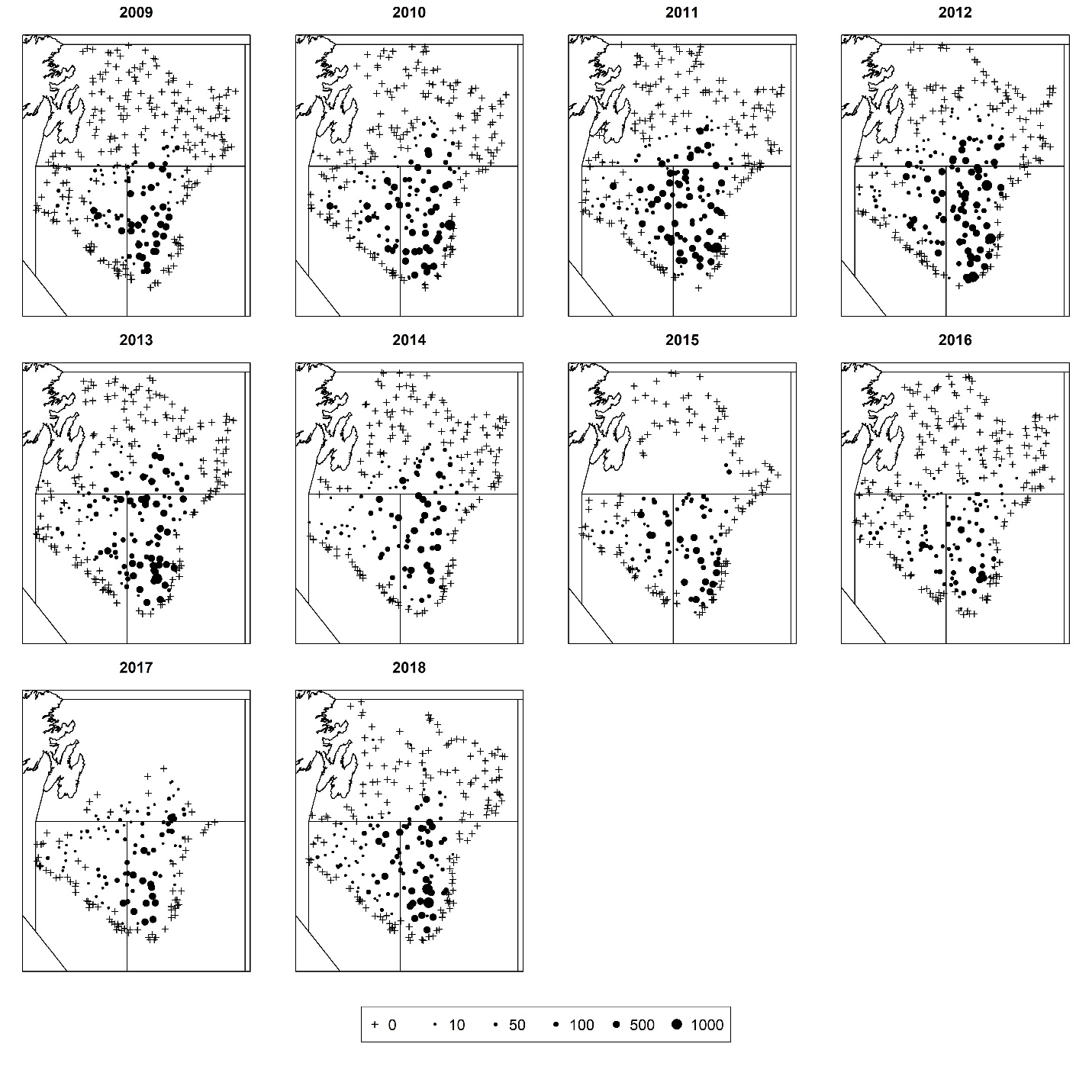


Figure 22. Observed survey catches for yellowtail flounder in NAFO divisions 3LNO from 2009 – 2018. No catch is denoted as a plus symbol and positive catches (biomass [kg]) are shown as points that increase in size with increased biomass caught. Figure made using the same software as Figure 20.


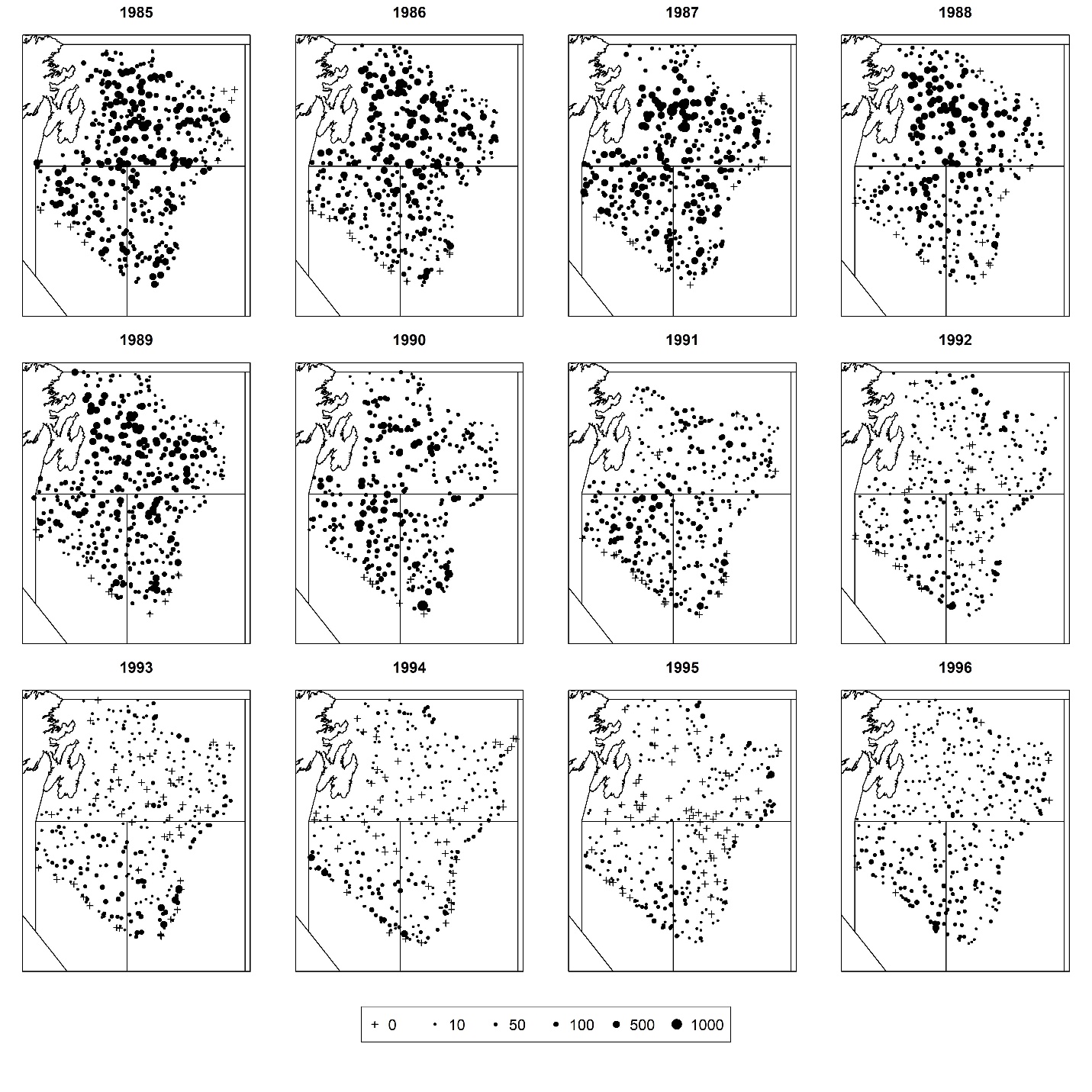


Figure 23. Observed survey catches for American plaice in NAFO divisions 3LNO from 1985 – 1996. No catch is denoted as a plus symbol and positive catches (biomass [kg]) are shown as points that increase in size with increased biomass caught. Figure made using the same software as Figure 20.


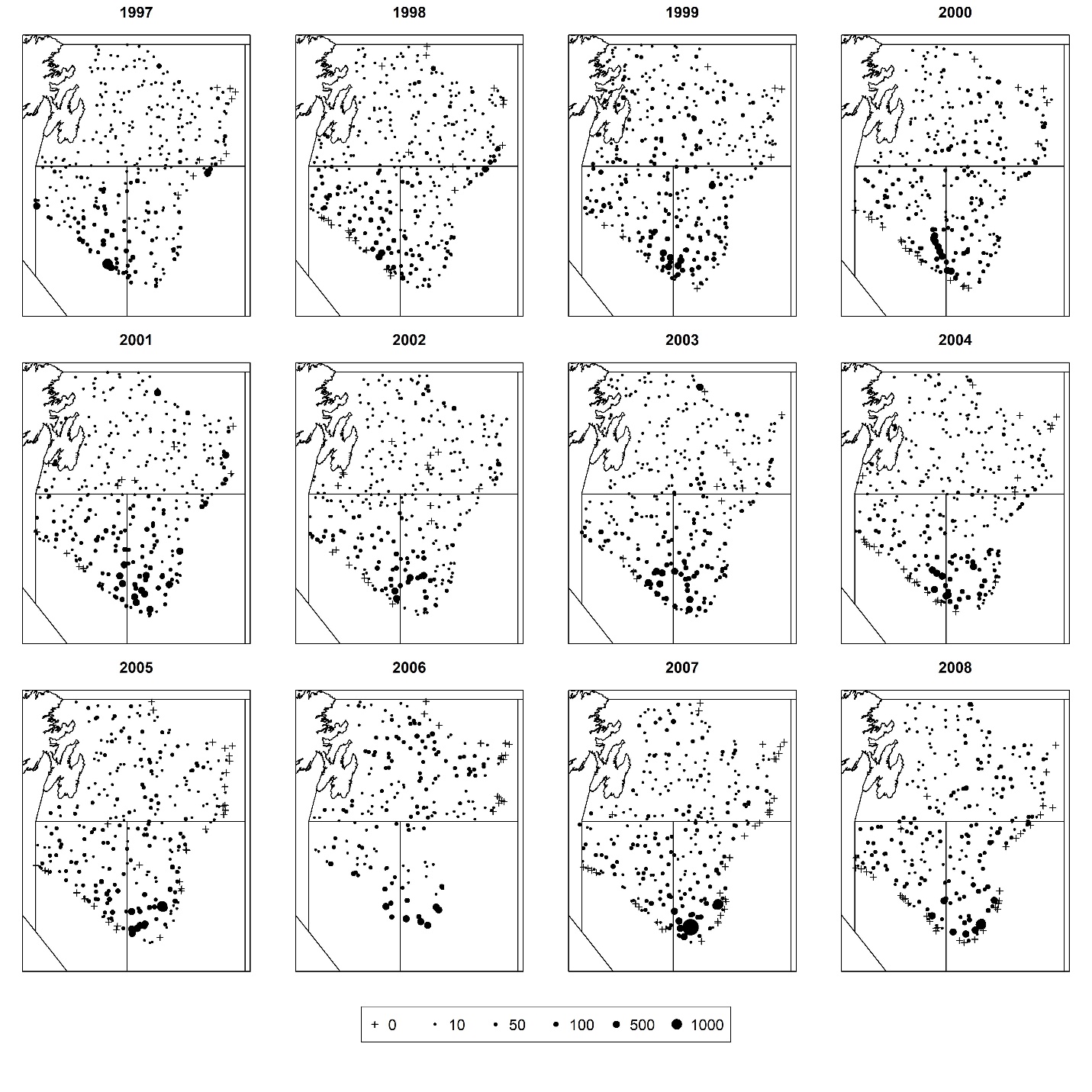


Figure 24. Observed survey catches for American plaice in NAFO divisions 3LNO from 1997 – 2008. No catch is denoted as a plus symbol and positive catches (biomass [kg]) are shown as points that increase in size with increased biomass caught. Figure made using the same software as Figure 20.


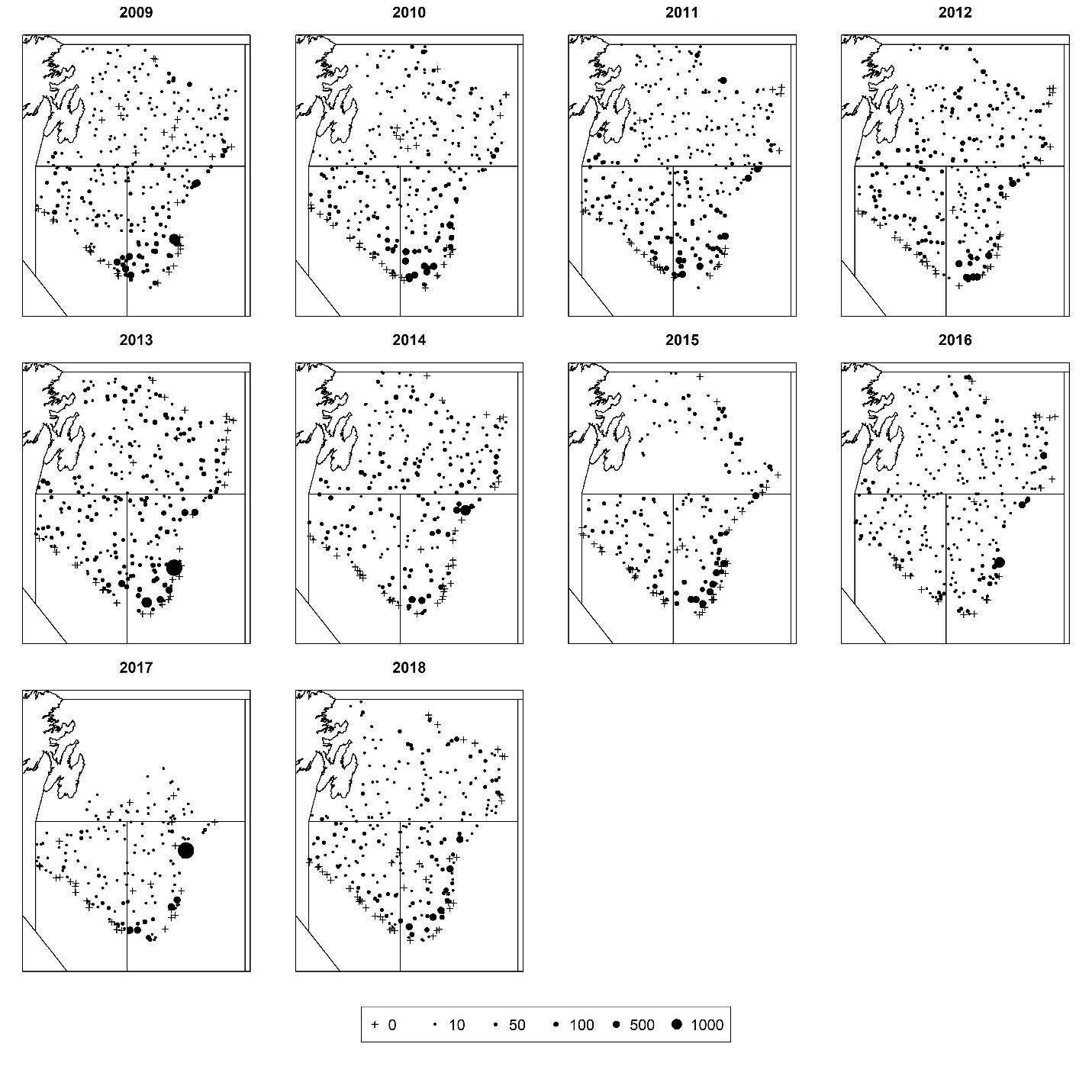


Figure 25. Observed survey catches for American plaice in NAFO divisions 3LNO from 2009 – 2018. No catch is denoted as a plus symbol and positive catches (biomass [kg]) are shown as points that increase in size with increased biomass caught. Figure made using the same software as Figure 20.


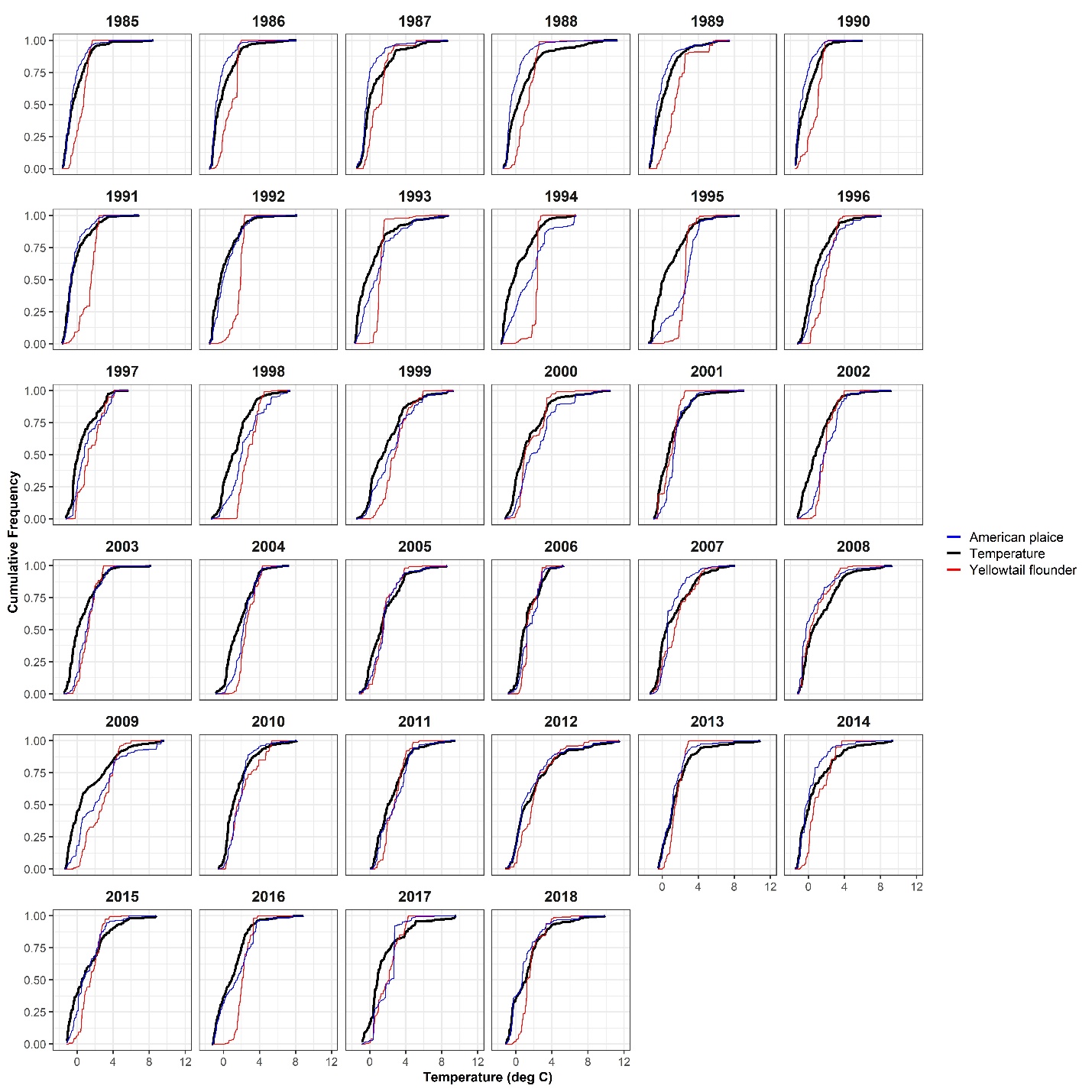


Figure 26. Cumulative distribution functions for the observed (raw data) temperatures (black lines), yellowtail flounder (red lines), and American plaice (blue lines) during surveys on the Grand Bank from 1985 – 2018. The curves were made using the methods described in Perry & Smith (1994)^13^. Figure made using R (version 3.6.2. https://www.r-project.org/)^1^ and the packages sp^2^ and ggplot2^8^.


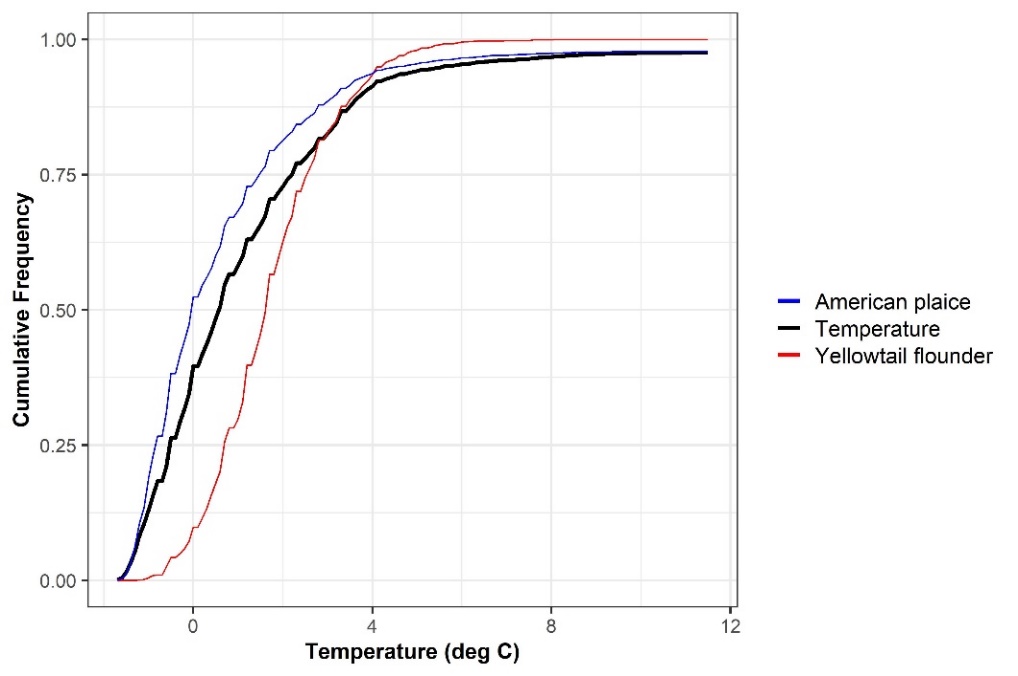


Figure 27. Cumulative distribution functions for the observed (raw data) temperatures (black lines), yellowtail flounder (red lines), and American plaice (blue lines) aggregated across all years (1985 – 2018). Differences in survey design and completeness over time (number of strata/strata sampled) resulted in temperature and American plaice curves not reaching one. Figure made using the same methods and software as Figure 26.


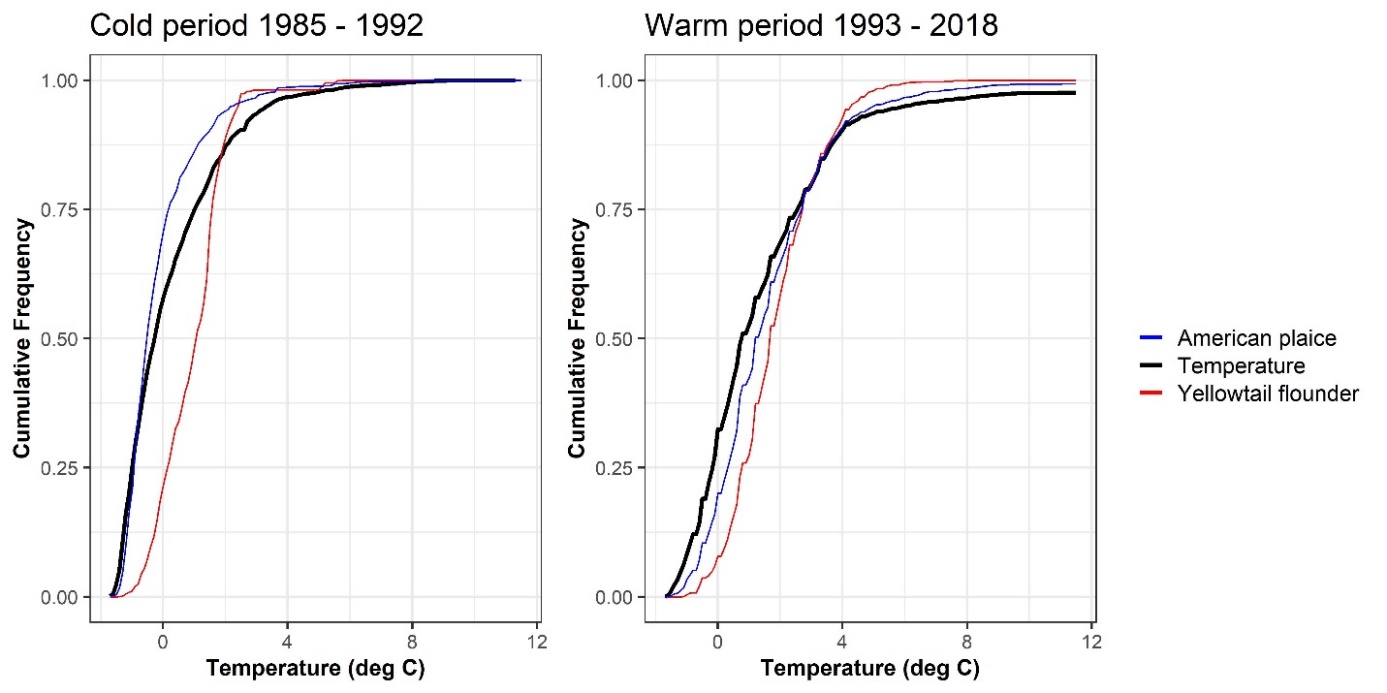


Figure 28. Cumulative distribution functions for the observed (raw data) temperatures (black lines), yellowtail flounder (red lines), and American plaice (blue lines) aggregated from 1985-1992 and 1993-2018 to represent differences in thermal preference before and after the cold period. Differences in survey design and completeness over time (number of strata/strata sampled) resulted in temperature and American plaice curves not reaching one in the warm period. Figure made using the same methods and software as Figure 26.


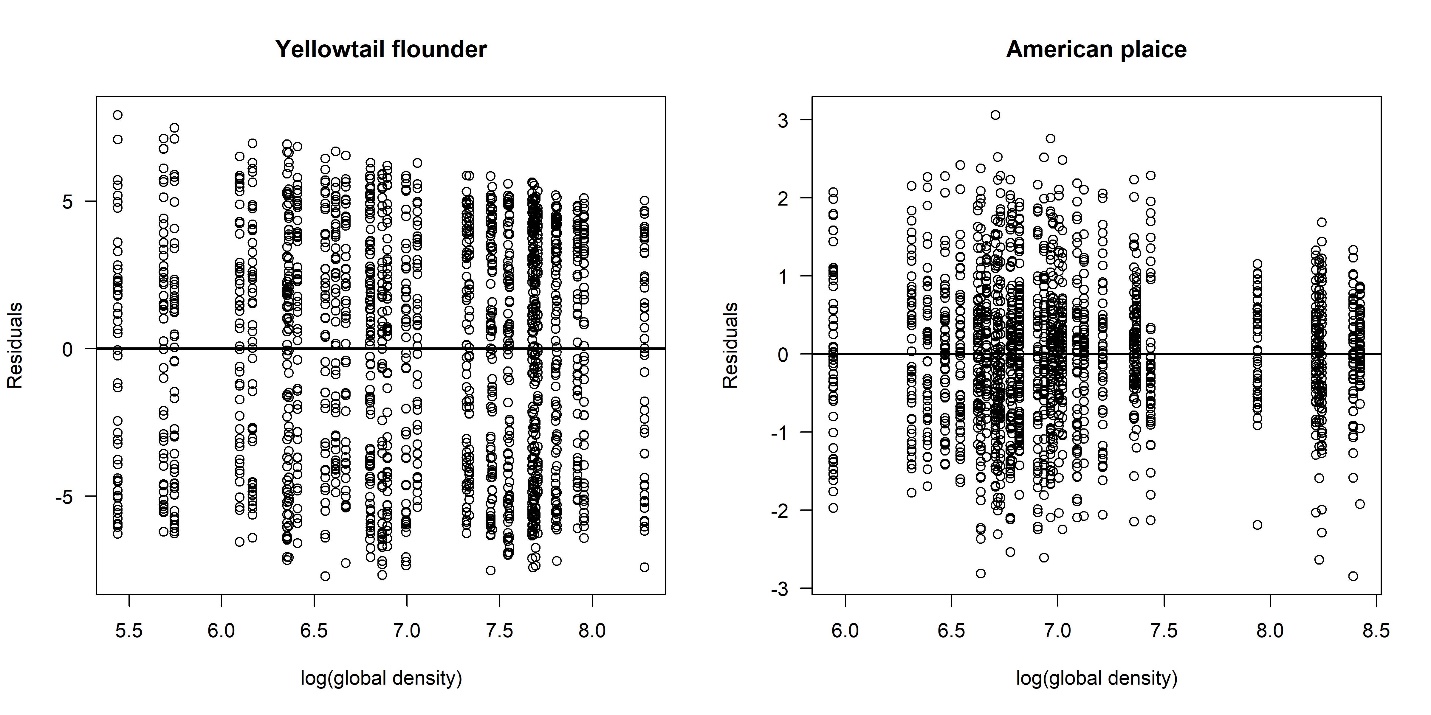


Figure 29. Relationship between the residuals from the linear model and the log of global density for yellowtail flounder and American plaice. The solid black line represents zero. Figure made using R (version 3.6.2. https://www.r-project.org/)^1^.


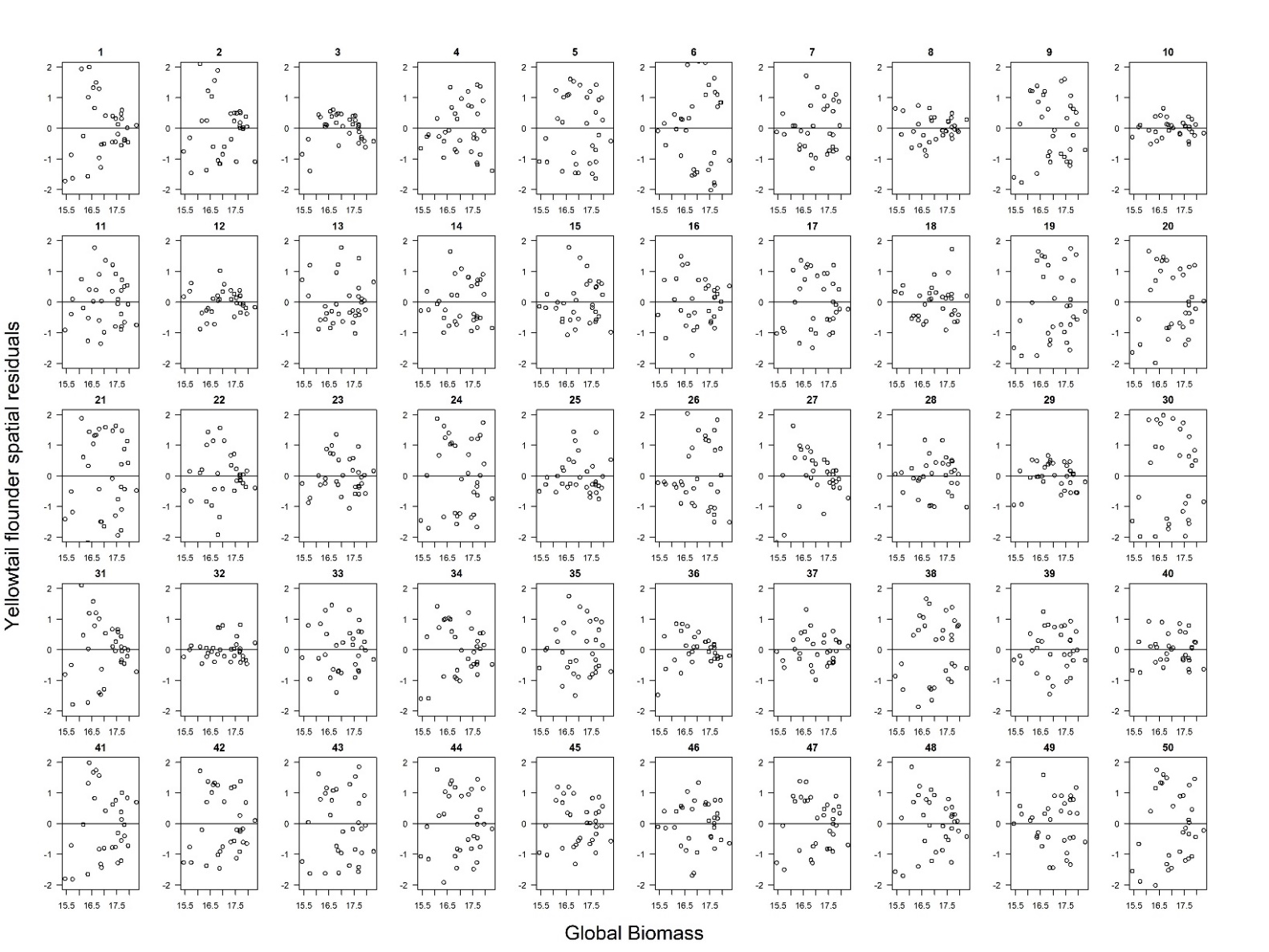


Figure 30. Relationship between the residuals from the yellowtail flounder mixed-effects model at all 50 knots (panels) and the yellowtail flounder global biomass in each year. The solid black line represents zero. Figure made using R (version 3.6.2. https://www.r-project.org/)^1^.

**References**

1. R Core Team. R: A language and environment for statistical computing. (2018).

2. Pebesma, E. & Bivand, R. Classes and methods for spatial data in R. (2005).

3. Hijmans, R. J. raster: Geographic Data Analysis and Modeling. (2016).

4. Bivand, R. & Rundel, C. rgeos: Interface to Geometry Engine - Open Source ('GEOS’). (2019).

5. Neuwirth, E. RColorBrewer: ColorBrewer Palettes. (2014).

6. Nychka, D., Furrer, R., Paige, J. & Sain, S. “fields: Tools for spatial data.” (2017) doi:https://doi.org/10.5065/D6W957CT.

7. Thorson, J. T. & Barnett, L. A. K. Comparing estimates of abundance trends and distribution shifts using single- and multispecies models of fishes and biogenic habitat. *ICES J. Mar. Sci.* **74**, 1311–1321 (2017).

8. Wickham, H. ggplot2: Elegant Graphics for Data Analysis. (2009).

9. Wheeland, L., Dwyer, K., Morgan, J., Rideout, R. & Rogers, R. Assessment of American Plaice in Div. 3LNO. **NAFO SCS D**, 77 (2018).

10. Parsons, D. M., Morgan, M. & Rogers, R. *Assessment of yellowtail flounder in NAFO divisions 3LNO using a new stock production model in a Bayesian framework*. (2018).

11. Pebesma, E. J. Simple Features for R: Standardized Support for Spatial Vector Data. *R J.* **10**, 439–446 (2018).

12. Bivand, R., Keitt, T. & Rowlingson, B. rgdal: Bindings for the ‘Geospatial’ Data Abstraction Library. (2019).

13. Perry, R. I. & Smith, S. J. Identifying Habitat Associations of Marine Fishes Using Survey Data: An Application to the Northwest Atlantic. *Can. J. Fish. Aquat. Sci.* **51**, 589–602 (1994).
